# Identifying subgroups of nonsuicidal self-injury: A systematic review

**DOI:** 10.1371/journal.pmen.0000291

**Published:** 2025-04-21

**Authors:** Kaylee P. Kruzan, Eva Hentges, Israel Ramirez, Jason J. Washburn

**Affiliations:** 1 Department of Preventive Medicine, Northwestern University Feinberg School of Medicine, Chicago, Illinois, United States of America; 2 Department of Psychiatry and Behavioral Sciences, Northwestern University Feinberg School of Medicine, Chicago, Illinois, United States of America; Universidad del Valle, COLOMBIA

## Abstract

Nonsuicidal self-injury (NSSI) is a complex behavior, and its presentation is marked by significant heterogeneity, complicating efforts to identify and intervene. In this study, we sought to systematically review studies that used data-driven classification methods to identify NSSI subgroups. We searched PubMed, MEDLINE, EMBASE, PsycINFO, Web of Science, and Scopus. Article were included if they: (1) focused on NSSI, relative to suicidal self-injury, (2) were published in a peer-reviewed journal, (3) in English language, (4) applied data-driven classification methods to identify subgroups of NSSI and (5) provided details about building the analytical models. Two investigators independently screened abstracts and full-text articles and assessed study quality. In total, 26 articles published between 2008 and 2023 were identified by the search. Studies included data from 7,388 individuals with a history of NSSI and identified 94 subgroups. Many subgroups were defined by NSSI characteristics including NSSI methods, NSSI functions, and lifetime frequency. Others focused on emotion regulation, social competencies, or relationship quality. The quality of study designs and reporting varied. Understanding heterogeneity within NSSI through these subgroups can help inform treatment and intervention personalization.

## Introduction

Nonsuicidal self-injury (NSSI) refers to self-inflicted damage of body tissue without suicidal intent and for purposes not culturally or socially sanctioned [1]. The behavior is prevalent among adolescent (17-27%) and young adult (13%) populations [2]. Among populations who experience emotional disorders, NSSI is more common [3]. Of even greater concern, however, is that NSSI frequently co-occurs with suicidal ideation and is a leading risk factor for future suicidal thoughts and behaviors [4,5]. It is estimated that between 50%–75% of those engaging in NSSI go on to make a suicide attempt in their lifetime [6]. Risk is believed to increase with repeated episodes of NSSI due to habituation to pain and a diminished instinct for self-preservation – both of which are factors that increase capability of suicide in the Interpersonal-Psychological Theory of Suicide [7]. Given the prevalence and associated risk, understanding and addressing NSSI is a public health priority.

NSSI is a complex behavior, and its presentation is marked by significant heterogeneity which complicates efforts to identify when it is necessary to intervene, and how best to intervene. Individuals that engage in NSSI can vary in the methods they employ, the frequency of engagement, the severity of injury, and the reasons for, or functions (antecedents and consequences that produce or maintain the behavior) of, the behavior [8]. Also, while NSSI can occur independent of other diagnoses, it is commonly comorbid with other mental health conditions like depression, eating disorders, and borderline personality disorder (BPD) [9,10]. Given this, effective intervention for NSSI must consider the behavior, the functions that it serves, as well as comorbid symptomology that may contribute to overall distress and functional impairment.

Yet, many individuals that engage in NSSI do not get effective treatment in part because of low rates of disclosure rates and internalized stigma [11,12], and in part due to widespread misunderstandings and misperceptions of NSSI [13]. The literature has shown that professionals in treatment settings often feel underprepared to respond to individuals that present with NSSI, harbor unhelpful misperceptions, and would benefit from additional training [14–17]. Consequently, a better understanding of the complex presentations of NSSI can not only help to identify and intervene on NSSI but also to increase confidence and competency for individuals in positions to intervene.

Investigations of NSSI heterogeneity have typically sought to group individuals based on characteristics of NSSI using latent class analysis or cluster analysis (e.g., k-means). These techniques derive subgroups of individuals with similar characteristics from larger heterogeneous groups. For example, Klonsky and Olino (2008) employed latent class analysis to explore NSSI presentations among college students [18]. They found four subgroups: (1) an experimental NSSI subgroup that engaged in NSSI on a few occasions; (2) a mild NSSI group which engaged in more frequent NSSI, and reported more BPD symptomology; (3) a multiple functions/anxious subgroup which endorsed a variety of NSSI methods, high rates of social and automatic or intrapersonal functions, and high anxiety; and (4) an automatic/intrapersonal functions/suicidal subgroup that consisted predominantly of individuals that cut themselves in private and reported automatic/intrapersonal functions. While some studies have replicated these subgroups [19], others have identified different subgroups. For example, He et al., 2023 used latent class analysis and found two subgroups of treatment-engaged adolescents that differed in their rates of suicidal ideation [20]. The subgroup with high suicidal ideation reported more frequent NSSI, endorsed more methods, and more physical pain. By contrast, the low suicidal ideation NSSI subgroup reported the use of NSSI methods with potential for less tissue damage.

While numerous studies have used data-driven methods, like latent class analysis and cluster analysis, to identify NSSI subgroups, a systematic review of these studies is needed to summarize the existing data and to identify possible convergence. Ultimately, understanding nuanced presentations of NSSI could facilitate more efficient and efficacious assessment and guide the development of personalized treatment and intervention.

## Review objective

The objective of this systematic review is to identify and review empirical research that has used data-driven classification methods to identify subgroups among individuals that engage in NSSI. We explore data-driven methods, approaches that apply statistical techniques to group data based on observed patterns, rather than more traditional methods of grouping individuals (e.g., experience or theory-driven subgrouping) since these methods allow researchers to explore the structure and relationships of many symptom characteristics (or features) with fewer a priori assumptions about subgroups. We use the term “subgroup” to refer to distinct groups of people with NSSI experience. Our research questions are: What subgroups exist in studies that use data-driven methods to group individuals reporting a lifetime history of NSSI? Do these subgroups (and the number of subgroups) differ by sample characteristics (age, geography, and type of analysis)? What key covariates and correlates are related to the NSSI subgroups? In answering these research questions, we ultimately aim to summarize existing literature, identify research gaps and opportunities, and discuss implications for treatment and intervention.

## Materials and methods

The methods for this review conform to the Prepared Items for Systematic reviews and Meta-Analysis (PRISMA) guidelines and the PRISMA-P checklist [21] (See S1 PRISMA Checklist). The protocol for the systematic review was registered on PROSPERO (CRD42024505683). All methods and analyses were determined a priori.

### Information sources and search strategies

The search strategy was formulated and refined by all members of the research team. Preliminary searches were conducted in PubMed and PROSPERO to identify systematic or scoping review protocols or publications that addressed our primary review question. One systematic review focused on NSSI typologies was in progress at the time of our review, and has subsequently been published [22]. This review has a more narrow focus than the present review as it excluded studies that examined presentations of NSSI within other clinical or psychiatric populations. Given that NSSI is highly comorbid with other mental health conditions, and comorbidity can impact treatment, we feel our review builds upon and extends their findings in a way that can inform treatment personalization. On February 4th, 2024, searches were run in the following six databases: PubMed, MEDLINE, EMBASE, PsycINFO, Web of Science, and Scopus. The search looked for medical subject headings and keywords related to NSSI and data-driven classification methods. A full list of search strategies and terms used is provided in supplemental materials (S1 Appendix). Reference lists were evaluated for additional articles not identified by the search strategy.

### Search inclusion and exclusion criteria

Studies were included if they were: (1) published in a peer-reviewed journal, (2) in English language, (3) applied data-driven methods to identify subgroups in a sample of individuals with a lifetime history of NSSI and (4) provided sufficient detail about the analytical models used to derive subgroups such that it could allow for comparability across studies. Here, sufficient detail meant that articles identified the variables that were entered into the model to derive subgroups as this detail is necessary for the interpretability of subgroups and subgroup descriptions. We included specific terms for latent class and cluster analysis in our search strategy because these are common data-driven methods for identifying typologies in the clinical and NSSI literatures. Other data-driven methods like random forest modeling are well-suited to prioritize specific features that are most influential in modeling but often produce complex (or black box) results that are difficult to interpret. Because a main aim of this work was to describe what factors were used to derive subgroups and differences in these factors across subgroups, we did not include these analyses in our review. There were no further exclusion criteria based on participant or sample characteristics. Publications available between the start date of each database to the time of the final search date were included.

### Search and review strategy

After running the search, a pilot screen of 50 random articles from the dataset was initially performed to clarify study inclusion and exclusion criteria and ensure consistency in the application of these criteria. Covidence systematic review software was used to manage the screening and review process once criteria were set [23]. Three reviewers (KPK, EH, IR) screened article titles and abstracts to determine whether articles should advance to full-text screening or be excluded using a series of predetermined criteria. Each article was reviewed independently by a minimum of two reviewers. Criteria were as follows: The title or abstract must (1) mention “self-injury” or “self-harm,” (2) derive subgroups, classes, groups, or profiles, (3) differentiate between suicidal and nonsuicidal self-injury (if no, these were tagged with “type 1” [24,25] and if unclear, these moved to full text review), and (4) focus on a sample in which all participants reported current or lifetime NSSI (if no, these were tagged with “type 2” [26,27], and if unclear, these were moved to full text review). See S2 Appendix for decision tree. Differences regarding inclusion and exclusion were resolved through discussion and consensus among all reviewers (KPK, EH, IR).

At the full-text review stage, articles were systematically excluded in a stepped fashion based on the same criteria as in the title and abstract screening. Throughout the full-text review process, discrepancies were resolved via consensus discussions among at least two reviewers. If consensus could not be reached, the third reviewer was brought into the discussion to adjudicate. For data extraction, a pilot was run on an initial sample of three studies to ensure agreement among extractors, resulting in refinement of the extraction table. Extraction was completed by two reviewers (EH, IR) with a final review of all extraction results done by the first author (KPK). Data extraction categories included: (1) study design, (2) population (e.g., clinical, community, university, online), (3) geographic region, (4) analysis method, (5) features (indicators used to derive subgroups), (6) covariates/correlates, (7) primary outcomes/auxiliary variables, (8) measure of NSSI, (9) number of subgroups, (10) subgroup percentages, (11) subgroup descriptions. All data elements were extracted for all studies. In cases where data elements were unclear or missing, a plan was in place to contact the author of the study. However, these data elements were present for all studies at the full text review stage (See S4 Table for data extraction and S5 Table for articles excluded at full text stage).

### Data synthesis and appraisal

We conducted a qualitative synthesis to describe the included studies and relationships between these studies in text and table format. To understand the level of detail on the analytical procedures used to derive groups in included studies, two independent reviewers used an adapted version of the Guidelines for Reporting on Latent Trajectory Studies (GRoLTS) checklist [28] for LCA studies, consistent with prior reviews [29]. We modified the original version by dropping items that were specific for longitudinal or trajectory analysis, but not relevant for the analyses in our review, resulting in a 17-item assessment tool. The use of GRoLTS deviates from what was registered in PROSPERO; however, the GRoLTS facilitated a more meaningful assessment of the clarity and transparency of reporting, which is a necessary precursor to an evaluation of confidence and generalizability of findings. The checklist includes items such as “Is entropy reported?,” “Reports missing data mechanism?,” and “Distribution of the observed variables reported?” Studies with fewer items checked lacked details that would be necessary to determine study quality. Disagreements in checklist completion were resolved by discussion and consensus.

## Results

**Fig 1:**
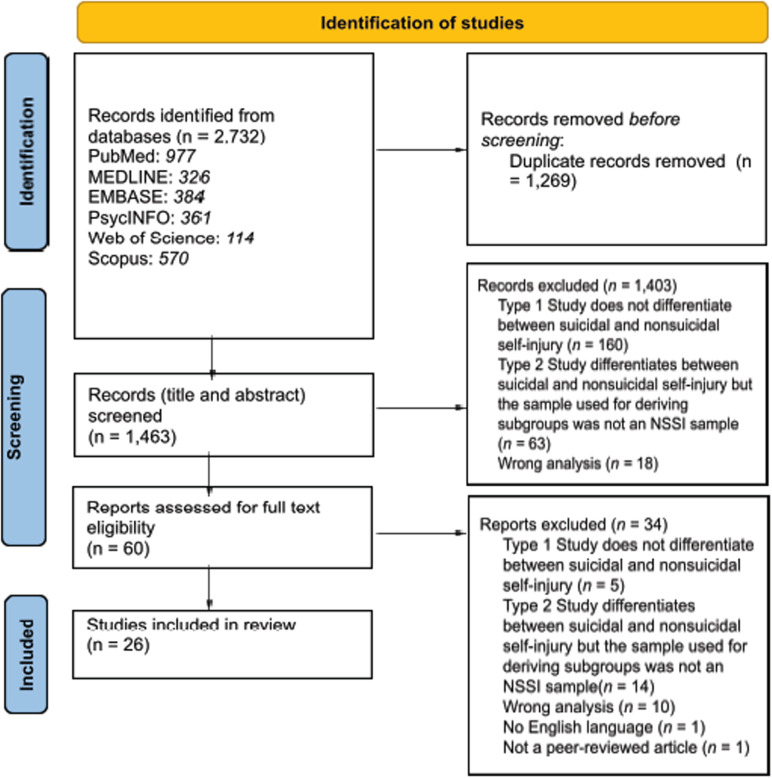
PRISMA flow diagram.

A total of 2,732 references were identified across all databases. After deduplication, 1,463 articles remained and were subjected to title and abstract screening, 1,403 were excluded, and the full texts of 60 studies were downloaded and screened for inclusion and exclusion. Of these 60 studies, 34 were excluded, leaving 26 articles for this review. Studies included data from 7,388 individuals with a history of NSSI and identified 94 subgroups. Fig 1 provides an overview of the article selection process.

### Study characteristics

Table 1 presents a general description of studies. All studies were published between 2008 and 2023. Eight studies were based in the U.S., with three from Australia, three from Canada, two from China, two from Italy, and one each from Germany, Singapore, Turkey, India, Korea, Budapest, Netherlands, and Portugal. One study ran an LCA on two separate samples [30]. Most samples were drawn from universities (*n* = 11), followed by clinics (*n* = 7), communities (*n* = 5) and primary or secondary schools (*n* = 4). Sample sizes ranged from 60 [31] to 672 participants [32]. Across studies, participant ages ranged between 9-56 years old, with eight studies focused on adolescents, seven studies focused on young adults, six studies focused on combined samples of adults and five studies focused on all ages. NSSI behavior was assessed through a variety of measures including the Inventory About Statements of Self-Injury (ISAS; *n* = 10), Diagnostic and Statistical Manual – Version 5 (DSM 5; *n* = 8), Diagnostic and Statistical Manual – Version 4 (DSM 4; *n* = 3), Deliberate Self-Harm Inventory (DSHI; *n* = 3), and Ottawa Statements about Self-injury (OSI; *n* = 2). Most studies included samples with a lifetime history of NSSI (*n* = 10), while others focused on more recent injury, including the past 12 months (*n* = 4), six months (*n* = 2), three months (*n* = 1), and one month (*n* = 1). The remaining studies reported that they included individuals that engaged in NSSI without specifying a time period (*n* = 8).

**Table 1:** Study characteristics*.*

Year	Author	Country	Population	Population description	Age M(SD); Range	N	NSSI measure^a^
2023	Gray	Australia	University and Community	Community members and university students who reported a lifetime history of NSSI.	21(4.43); 17-39 years old	224	ISAS
2023	He	China	Clinical	In- and out-patient adolescents who had self-injured in the past year.	14.7(1.6); 12-18 years old	326	DSM-5
2023	Kim	Korea	Community	Adults who repeatedly engaged in self-harm behavior within the last six months.	22.47(3.35); 19-35 years old	60	SHI/ DSM-5
2023	deNeve-Enthoven	Netherlands	Primary/ Secondary School	High-risk adolescents reporting lifetime history of NSSI.	15.0(.09); 12.9-18.1 years old	322	ISAS
2023	Yan	China	Clinical	Psychiatric inpatients who reported lifetime history of NSSI.	18.89(2.49); 18-25 years old	190	DSM-5
2022	Dixon-Gordon	United States	Community	Adolescents and young adults from online forums (Sample 1) or the community (Sample 2) that reported NSSI behavior in the past three months.	Sample 1: 21.18(4.15); 15–35 years old Sample 2: 23.84 (4.8); 15-35 years old	Sample 1: 155 Sample 2: 127	DSHI
2022	Gonçalves	Portugal	Clinical	Outpatients with eating disorders who reported a lifetime history of NSSI.	26.42(9.35); 14-55 years old	73	DSM-5
2022	Mürner-Lavanchy	Germany	Clinical	Outpatients with NSSI disorder.	14.95(1.46); 12-17 years old	240	DSM-5
2022	Raffagnato	Italy	Clinical	Young inpatients on a neuropsychiatry unit in northern Italy with a history of NSSI.	14.4(1.59); 9-17 years old	94	DSM-5
2022	Reinhardt	Budapest	Primary/ Secondary School	Adolescents who identified as engaging in at least one episode of NSSI in the past month.	16.72(1.41); 14-20 years old	322	ISAS
2022	Sack	Canada	Clinical	Youth admitted for psychiatric hospitalization who reported engaging in NSSI.	14.25(1.76); 10-17 years old	68	ISAS
2021	Christoforou	Australia	University	Undergraduate students who reported a lifetime history of NSSI	21.5(5.3); 17-56 years old	270	ISAS
2021	Goddard	Australia	University	University students who had engaged in NSSI.	21.59(5.43); 17-56 years old	236	ISAS
2021	Guérin-Marion	Canada	University	University students who had engaged in NSSI within the past year.	18.77(1.42); 17-25 years old	479	OSI
2021	Singhal	India	Community	Graduate and post-graduate students from 19 educational institutes from the city of Bengaluru, India, who had engaged in NSSI in the past year	20.69(1.72); 18-25 years old	353	DSM-5
2020	Case	United States	University	University students with a history of engagement in NSSI.	20.4 (3.3); 17-23 years old	359	DSHI
2020	Shahwan	Singapore	Clinical	Outpatients attending a tertiary psychiatric hospital in Singapore who reported at least one NSSI behavior within the last year.	22.0 (5.58); 14-35 years old	235	DSM-5
2019	Gargiulo	Italy	Primary/ Secondary school	Italian high school students reporting a lifetime history of NSSI.	14.6(.9)	108	ISAS
2019	Peterson	United States	University	Undergraduate college students from the southeastern U.S. endorsing lifetime NSSI.	21.33(4.16); 18-51 years old	326	DSHI
2016	Martin	United States	University	Undergraduate students endorsing at least 1 NSSI behavior in the past 6 months.	19.37(1.5); 17-25 years old	264	OSI
2015	Somer	Turkey	Primary/ Secondary School	High school students reporting a lifetime history of NSSI.	16.8(1.26)	519	ISAS
2015	Vaughn	United States	Community	A nationally representative sample of non-institutionalized U.S. residents engaging in NSSI.	18 and olders*	672	DSM-4
2013	Hamza	Canada	University	First-year undergraduate students from a mid-sized Canadian university reporting a lifetime history of NSSI.	19.11(1.05); 17-19 years old	439	ISAS
2012	Bracken-Minor	United States	University	College students and internet users with a lifetime history of NSSI.	22.85(6.17); 18-56 years old	440	ISAS
2008	Klonsky	United States	University	College students who endorsed at least one NSSI behavior.	18.5(1.2)	205	DSM-4
2008	Whitlock	United States	University	Undergraduate and graduate students from two northeastern universities reporting 2 or more NSSI episodes.	18-24 years old	282	DSM-4

*Note.* Table 1 is organized by year, followed by alphabetical order corresponding with the authors’ last name.

^a^NSSI measures include: Deliberate Self-Harm Inventory (DSHI), The Non-Suicidal Self-Injury Assessment Tool (NSSI-AT), Self-Injurious Thoughts and Behaviors Interview (SITBI), Alexian Brothers Assessment of Self-Injury (ABASI), Functional Assessment of Self-Mutilation (FASM), Inventory of Statements (ISAS), Diagnostic and Statistical Manual-5 (DSM-V), Diagnostic and Statistical Manual-4 (DSM-IV), Ottawa Self-Injury Inventory (OSI); N reflects the number of individuals in the subgroup analysis.

Table 2 presents additional details about the analytical approach, models, and 94 subgroups identified across studies. The majority of studies derived subgroups using latent class analysis (*n* = 14), followed by cluster analysis (*n* = 8), latent profile analysis (*n* = 3) and latent variable mixture modeling (*n* = 1). All studies identified between 2 and 5 subgroups, with 4 being the most frequent number of subgroups. There was significant variability in what indicator variables were used to derive models, and subsequently what variables were explored across groups (S1 Table). Most studies included NSSI characteristics as model features (*n* = 17), with methods (*n* = 13), functions (*n* = 10) and lifetime frequency (*n* = 8) being most common. The remaining studies used other psycho-social variables as indicators and explored NSSI characteristics as outcomes (*n* = 8). Only one study included NSSI characteristics and other psycho-social competencies and behaviors together as indicators [33]. Almost all studies used the derived subgroups as independent variables predicting other relevant outcomes; however two studies used subgroups as dependent variables [34,35].

**Table 2:** Model features, subtypes, findings.

Year	Author	Analysis	Model Features/Indicators	# of subtypes	Subtype Descriptions	DV or IV	Selected Findings
2023	Gray	Latent profile analysis	Extent to which one has wanted to and not wanted to self-injure over their lifetime (2 items, 10-point scale)	4	1. Highly ambivalent (*n* = 30; 13.4%) 2. Avoid (*n* = 39; 17.4%) 3. Approach (*n* =70; 31.3%) 1. Moderately ambivalent (*n* = 85; 37.9%)	DV: Tendency to approach/avoid NSSI, NSSI characteristics and functions, Personality, Reasons to stop self-injury, Difficulties in emotion regulation, Psychological distress, NSSI-related outcome expectancies, Self-Efficacy to Resist NSSI	• Avoid: Most likely to not want to self-injure in past month; Older; Higher extraversion; Reported more reasons to stop • Approach: Least likely to not want to self-injure; Younger; Younger age of onset; Higher neuroticism; More likely to report intrapersonal functions; Fewer reasons to stop; Higher addictive qualities; Less desire for change; More difficulties in emotion regulation; greater affect regulation expectancies; Less ability to resist. • Moderately Ambivalent: reported midway levels of wanting to self‐injure, and midway levels of not wanting to self‐injure throughout their lifetime • Highly Ambivalent: reported high levels of wanting to self‐injure, and high levels of not wanting to self‐injure throughout their lifetime.
2023	He	Latent class analysis	12 NSSI behaviors (binary; FASM), suicidal ideation and suicide attempt (binary)	2	1. High suicidal ideation NSSI (*n* = 129, 39.6%) 2. Low suicidal ideation NSSI (*n* = 197, 60.4%)	DV: Suicide attempt, Depression (PHQ9), Self-injury functions (FASM), Resilience (CD-RISC-10), Alexithymia (TAS), Peer victimization (MPVS) Social support (Multidimensional Scale of Perceived Social Support; MSPSS).	• High suicidal ideation NSSI: Lower perceived social support; girls, left-behind experience, single parent family, peer victimization and depression, relative to Low suicidal ideation NSSI.
2023	Kim	Latent class analysis	21 NSSI behaviors (SHI)	2	1. Substance abuse and suicide attempt (*n* = 30; 50%) 2. Cutting and scratching (*n* = 30; 50%)	DV: Suicide attempt (SASII), Aggression (BPAQ), Symptoms of BPD (PAI-BOR), Emotion Regulation (DERS), Symptoms of PTSD (IES-R) EMA: Emotions, thoughts, and urges related to self-harm and suicide.	• Substance use and suicide attempt: More likely to report psychiatric histories, lifetime suicide plans and attempts, lifetime number of suicide attempts, the number of diagnoses and medication history. Endorsed higher BPD symptoms, aggression, posttraumatic symptoms and difficulties in emotion regulation, relative to Cutting and scratching. • On EMAs, Substance use and suicide attempts: Higher anger towards others, feelings of rejection, loneliness, and helplessness and more likely to report urges to self-harm, relative to Cutting and scratching
2023	deNeve-Enthoven	Latent class analysis	Lifetime NSSI frequency, lifetime number of NSSI methods, NSSI methods, NSSI urgency, pain during NSSI, lifetime suicidal ideation, lifetime suicide attempt (all categorical), and intrapersonal and interpersonal functions of NSSI (both continuous) (ISAS)	4	1. Low NSSI - Low suicidality (*n* = 108; 33.6%) 2. Moderate NSSI - Low suicidality (*n* = 95; 29.6%) 3. Moderate NSSI - High suicidality (*n* = 53; 16.5%) 4. High NSSI - High suicidality (*n* = 65; 20.2%)	IV: sex, age, ethnic background, household monthly income, non-verbal IQ score, internalizing and externalizing problems, family functioning, social support from respectively family, friends and significant others, and self-esteem	• High NSSI - High Suicidality: More likely to report household income in the 2400-4399 euros range, than Moderate NSSI - High Suicidality and significantly less often in the highest income level than in Moderate NSSI - Low Suicidality. • High NSSI-High Suicidality: More externalizing problems, lower self-esteem, and less social support from their family relative to Low NSSI - Low Suicidality and Moderate NSSI - Low Suicidality. • High NSSI - High Suicidality: Lower levels of social support from friends, relative to Low NSSI-Low Suicidality. • Moderate NSSI - Low Suicidality: Lowest levels of social support from a significant other. • Subtypes with high levels of suicidality (3 and 4) comprised a significantly larger proportion of girls and reported significantly higher levels of internalizing problems than classes with low levels of suicidality (1 and 2).
2023	Yan	Cluster analysis	NSSI functions: Emotional regulation, Social influence, Sensation seeking, and Anti-suicide (27 items; 5-point scale; OSI)	5	1. Typical NSSI (*n* =19; 10.0%) 2. Sensation Seeking NSSI (*n* = 47; 24.7%) 3. Social Influence NSSI (*n* = 22; 11.5%) 4. Anti-Suicide NSSI (*n* = 41; 21.5%) 5. Untypical NSSI (n = 61; 32.1%)	DV: Existing mental health disorders, Occasional or repeated NSSI	• Untypical NSSI: Reported more occasional NSSI. • Sensation Seeking: More repeated NSSI and greater proportion of depressive symptoms • Anti-Suicide NSSI: Greater proportion of borderline patients. • Social Influence NSSI: Greater numbers of ED patients.
2022	Dixon-Gordon	Latent profile analysis	The five NSSI motives (emotion relief, feeling generation, interpersonal communication, interpersonal, influence, and self-punishment; from QNSSI and SASII)	5/5	1. Low Interpersonal Motives (Sample 1: *n* = 65; 41.9%; Sample 2: *n* = 56; 44.1%) 2. Self-punishment/Interpersonal Motives (Sample 1: *n* = 47; 30.3%; Sample 2: *n* = 14; 11.0%) 3. Moderate Intra/Interpersonal Motives (Sample 1: *n* = 19; 12.3%; Sample 2: *n* = 23; 18.1%) 4. High Intra/Interpersonal Motives (Sample 1: *n* = 14; 9.0%; Sample 2: *n* =21; 16.5%) 5. Mainly Interpersonal Motives (Sample 1: *n* = 10; 6.5%; Sample 2: *n* = 13; 10.2%)	DV: Demographic variables (i.e., age, BIPOC status, sex), NSSI characteristics (i.e., NSSI frequency, versatility [number of different NSSI methods], medical severity), and measures of psychopathology (i.e., depression, BPD, emotion regulation difficulties).	Sample 1: • There were no significant differences between classes in terms of demographics, NSSI frequency (lifetime or past three-month), versatility, or medical severity or BPD symptoms • High Intra/Interpersonal Motives: More depression symptoms than the Moderate Intra/Interpersonal Motives. • High Intra/Interpersonal Motives: Greater emotion regulation difficulties than both the Low Interpersonal Motives and Moderate Intra/Interpersonal Motives. Sample 2 (longitudinal) • No relationship between overall NSSI and class membership. Significant effect of time. • Low Interpersonal Motives: Decreased NSSI frequency and versatility over time. • Self-punishment/Interpersonal Motives: Increased NSSI engagement over time.
2022	Gonçalves	Cluster analysis	Eating pathology (ED-15), emotion dysregulation (DERS) and negative urgency (UPPS-P)	3	1. Moderate severity (*n* = 29, 39.7%) 2. High severity (*n* = 29, 39.7%) 3. Low severity (*n* = 15, 20.5%)	DV: eating pathology (ED-15), emotion regulation (DERS) and negative urgency (UPPS-P), age, BMI, durations of ED or treatment, number of methods of NSSI, current and past NSSI	• Moderate Severity: Included participants with higher levels of eating pathology (when compared to sample mean). Lower emotion dysregulation and negative urgency than High Severity, but higher relative to Low Severity subtype. Most frequent diagnoses were AN restricting type, BN and OSFED. More participants with past NSSI, relative to current. • High severity: Highest scores in eating pathology, emotion dysregulation and negative urgency. Most frequent diagnoses were BN and AN restricting type. More participants with current NSSI. • Low severity: Lowest levels of eating pathology, emotion dysregulation and negative urgency. Most frequent diagnosis was AN restricting type. More participants with past NSSI, relative to current.
2022	Mürner-Lavanchy	Latent class analysis	Neurocognitive performance (processing speed, attention, learning, working memory, and executive function)	2	1. Class 1/Better neurocognitive performance (*n* = 176; 73.3%): NA 2. Class 2/Worse neurocognitive performance (*n* = 64; 26.67%)	DV: Clinical global impression, global functioning, NSSI behavior (method, behavior), BPD diagnosis and number of BPD criteria.	• The two classes did not differ on most clinical variables (clinical global impression, global functioning, NSSI behavior, BPD diagnosis and number of BPD criteria) except frequency of NSSI during the past month, with Class 1 reporting higher rates of NSSI.
2022	Raffagnato	Cluster analysis	Social problems, social competencies, and affective disorders (YSR Scales)	4	1. Moderate affective difficulties with discrepant social functioning: (*n* = 26; 27.6%) 2. Socio-affective impairment (*n* = 21; 22.3%) 3. Good socio-affective functioning (*n* = 24; 25.5%) 4. Low affective difficulties with discrepant social functioning: (*n* = 23; 24.4%)	DV: Onset of NSSI; Presence of suicidal ideation and attempt, emotional regulation.	• Socio-affective impairment: All patients presented with suicidal ideation and 42.9% attempted suicide. They had the most severe emotional dysregulation. • Low affective difficulties with discrepant social functioning: 82.6% reported suicidal ideation and 34.8% attempted suicide. Moderate impairment in emotional regulation function • Socio-affective impairment and Low affective difficulties with discrepant social functioning included more patients with recent NSSI onset, relative to others.
2022	Reinhardt	Latent class analysis	12 NSSI methods (binary, ISAS)	2	1. Severe/Multimethod NSSI (39%) 2. Mild/Moderate NSSI group (61%)	DV: Gender, age, NSSI motives, experience of pain during NSSI, injuring alone, urgency, desire to stop NSSI (ISAS), perfectionism and mental health (Adolescent Mental Health Continuum)	• Severe/Multimethod NSSI: Engaged in NSSI at high intensity and motivated mainly for intrapersonal reasons. More likely to report a lower level of mental health status. Experienced pain when injuring, urgency to engage in NSSI, more likely to injure alone • Age, gender, perfectionism, interpersonal motives and desire to stop did not differ between classes.
2022	Sack	Latent class analysis	12 NSSI functions (binary, DSHI)	2	1. Multiple Functions (*n* = 28; 46.6%) 2. Single/Avoidant Function: (*n* = 40; 58.8%)	DV: Gender, BPD symptomology (affective instability, negative relationships, and impulsivity), and NSSI features (frequency and hospitalization/severity) (DSHI).	• Multiple Functions: Greater affective instability, impulsivity, negative relationships, and greater engagement in NSSI. • No significant differences in age or gender by class.
2021	Christoforou	Cluster analysis	Emotion regulation (DERS total score, all subscales of the CERQ), Coping strategies (3 subscales of COPE), and Alexithymia (TAS-20 total score)	3	1. Considerate emotion difficulties (*n* = 98; 36.2%) 2. No emotion difficulties (*n* = 78; 28.8%) 3. Passive moderate emotion difficulties (*n* = 94; 34.8%)	DV: NSSI functions, Other dysregulated behaviors [Risky drinking (AusAUDIT), BPD symptoms (Borderline Symptom List), Disordered Eating (EAT-26)], Anxiety, Depression, Stress (DASS-21)	• Considerate Emotion Difficulties: More likely to have engaged in NSSI 5 or more times within the last year. Less likely to have not self-injured in the past year. More likely to report gender other than male or female. More likely to endorse affect regulation, self-punishment, antisuicide and sensation seeking functions. Scored significantly higher on all comorbid symptom scales. • No Emotion Difficulties: Older than those in Considerate Emotion Difficulties; less likely to have engaged in NSSI on 5 or more times in the past year; More males, relative to Passive Moderate Emotion Difficulties. • Passive Moderate Emotion Difficulties: More likely to report peer bonding function. More females.
2021	Goddard	Cluster analysis	Big Five personality traits (44 items, 5-point likert scale)	3	1. Disagreeable (*n* = 101; 42.7%) 2. Resilient (*n* = 80; 33.8%) 3. Dysregulated (*n* = 55; 23.3%)	DV: NSSI characteristics (ISAS), Experiential avoidance (Brief Experiential Avoidance Questionnaire), Alexithymia (Toronto Alexithymia Scale), Depression, Anxiety and Stress Scale, Emotion regulation (Cognitive Emotion Regulation Questionnaire)	• Dysregulated: More NSSI in the past year and greater likelihood of experiencing physical pain, higher affect regulation and self-punishment functions, higher alexithymia, anxiety, stress, and self-blame. • Resilient: More likely to endorse regulation techniques like acceptance, putting things into perspective, refocusing on planning, and positive reappraisal. • Disagreeable: High alexithymia and psychological distress.
2021	Guérin-Marion	Latent cluster analysis	Difficulties regulating positive and negative emotions (DERS; Mean Score) and Rumination (Ruminative Thought Style Questionnaire; Mean Score)	3	1. Average Difficulties (*n* = 227; 47.4%) 2. Dysregulated (*n* = 158; 33.0%) 3. Low Difficulties (*n* = 94; 19.6%)	DV: Self-reported quality of maternal and paternal relational experiences, and the frequency, time elapsed since onset, methods, functions, and addictive properties of NSSI	• Dysregulated: Higher unresolved attachment to fathers, higher perceived pressure from fathers, greater feelings of alienation from parents, and more pronounced histories of father antipathy during childhood/adolescence. Higher endorsement of external ER, internal ER, social influence, and sensation-seeking functions, as well as a higher endorsement of addictive features, relative to other clusters. Higher levels of father psychological control, father overprotection, maternal unresolved attachment, maternal pressure and number of NSSI methods, than Low Difficulties. • Average Difficulties: Higher reported father psychological control, father antipathy, father pressure, unresolved attachment to fathers, and general feelings of alienation from parents, higher internal ER functions, sensation-seeking functions, and addictive features than those in the Low Difficulties profile.
2021	Singhal	Cluster analysis	Severity (based on potential degree of tissue damage by method - minor, moderate, severe), frequency, diversification (# of NSSI methods endorsed), types of functions endorsed (interpersonal, intrapersonal, both), age of onset (all categorical, ISAS) and gender	5	1. Multimethod NSSI (*n* = 126; 35.70%) 2. Experimental NSSI (*n* = 74; 21%) 3. Female Minor NSSI (*n* = 64; 18.10%) 4. Male Minor NSSI (*n* = 46; 13.00%) 5. Exclusively severe NSSI (*n* = 43; 12.20%)	DV: Self-criticism (SRS), brooding-rumination (RRS-Brooding), emotion regulation difficulties (DERS), experiential avoidance (BEAQ) psychological distress (DASS-21), attachment style (ASQ) and perceived social support (ISEL-12)	• Multimethod NSSI: Higher psychological distress compared to the Experimental and Female Minor NSSI groups, but not significantly different from Male minor and Exclusively Severe NSSI groups. Higher difficulties in regulating emotions compared to the Experimental NSSI group, and the Male and Female minor NSSI groups, but not significantly different from the Exclusively Severe NSSI group. • The five clusters did not differ significantly on the measures of self-criticism, brooding-rumination, experiential avoidance, attachment style and perceived social support.
2020	Case	Latent class analysis	NSSI methods, number of methods, lifetime frequency rates, last year frequency rates, number of scars resulting from NSSI (DSHI), amount of pain experienced when self-injuring, and 13 functions of NSSI (categorical; ISAS)	4	1. Mild/experimental NSSI (39%) 2. Moderate NSSI (29%) 3. Moderate Multiple Functions NSSI (8%) 4. Severe NSSI (24%)	DV: Self-esteem (Rosenberg Self-Esteem Scale); Body Investment (Body Investment Scale); Social Appearance Anxiety Scale; Social Support and Belongingness (Multidimensional Scale of Perceived Social Support; MSPPS)	• Mild/experimental NSSI: More protective factors against NSSI, lower loneliness, scored significantly lower than all other classes on internalizing symptoms • Severe NSSI: Higher than other classes on all suicide variables (e.g., interrupted suicide attempts, aborted suicide attempts, suicidal preparatory behaviors, hospitalization by another person for suicide concern, suicidal attempt behavioral forecast, and suicidal ideation) excluding suicide planning, for which it scored significantly higher than the Mild/Experimental NSSI group, but did not differ significantly from others. More likely to endorse hurting themselves as a way to cope when feeling bad in the future.
2020	Shahwan	Latent class analysis	Frequency of NSSI (ordinal), length of contemplation before engaging in NSSI (ordinal), usage of more than three NSSI methods (binary), suicidal ideation (binary), some/often social-positive function (binary), some/often social-negative function (binary), some/often automatic-positive function (binary) and some/often automatic-negative function (binary) (FASM)	3	1. Experimental/Mild NSSI (*n* = 45; 19.2%) 2. Multiple functions NSSI/Low Suicide Ideation (*n* = 74; 31.5%) 3. Multiple functions NSSI/Possible Suicide Ideation (*n* = 116; 49.4%)	DV: Emotion regulation (DERS), childhood trauma (CTQ), depression (PHQ8), physical and mental health (Short Form 12)	• Multiple functions NSSI/Possible Suicide Ideation: Higher scores on impulse and awareness, relative to Experimental/Mild NSSI subtype • Multiple functions NSSI/Low Suicide Ideation: More likely to have low to moderate emotional abuse than Experimental/Mild NSSI subtype. • Experimental/Mild NSSI: Less likely to have depressive symptoms.
2019	Gargiulo	Cluster analysis	NSSI behavior, method, lifetime frequency, past year frequency, last episode, pain, loneliness, time between thought and behavior, attempted to stop (ISAS)	2	1. Repetitive NSSI (*n* = 41; 37.9%) 2. Episodic NSSI (*n* = 67, 62.0%)	DV: Body investment (the Body Investment Scale) and Emotion regulation (DERS)	• Repetitive NSSI: High affect regulation, self-punishment, and anti-suicide functions, high emotion regulation scores (inability to regulate emotions) • Episodic NSSI: High interpersonal influence, higher body protection scores
2019	Peterson	Latent class analysis	NSSI methods and frequency (DSHI) and emotion regulation difficulties (DERS)	4	1. Class 1/Moderate ER difficulties with high rates of cutting and burning (*n* = 8; 2%) 2. Class 2/High ER difficulties (*n* = 67; 21%) 3. Class 3/Moderate ER difficulties high rates of scratching/skin piercing (*n* = 12; 4%) 4. Class 4/Low ER difficulties (*n* = 231; 73%)	DV: Acquired capability and suicide attempt history (ACSS; SHBQ); impulsive behavior (UPPS); problematic alcohol use (AUDIT) disordered (EDE-Q)	• Class 1: Higher on acquired capability for suicide and higher levels of sensation seeking than Classes 3 and 4. • Class 2: Higher rate of suicide attempts than Class 4; higher levels of negative urgency than Classes 3 and 4 and higher levels of positive urgency, lack of pre-meditation, and lack of perseverance than Class 4. Higher levels of disordered eating than Class 4. • No differences were observed between classes for problematic alcohol use.
2016	Martin	Latent profile analysis	Perceptions of parent–child relationship quality (maternal lack of care, maternal control, paternal lack of care, parental control, trust in parent–child relationships, feeling alienated from parents, and relational trauma) (PBI, IPPA, AUAQ)	4	1. Negative - invalidating (*n* = 112; 42.4%) 2. Positive-moderate (*n* = 95; 35.9%) 3. Positive-idealistic (*n* = 35; 13.2%) 4. Negative-disturbed (*n* = 22; 8.3%)	DV: NSSI severity (age of onset, frequency, number of methods) and functions (OSI)	• Negative-disturbed: More internal ER functions than the Positive-idealistic and Positive-moderate profiles. Highest endorsement of external ER functions. More NSSI methods than Positive-idealistic and marginally more than Positive-moderate, no difference for Negative-invalidating. Earlier age of onset than Positive profiles. More likely to engage in NSSI more than 5 times in the past 6 months.
2015	Somer	Latent class analysis	12 NSSI behaviors, 2 functions (interpersonal and intrapersonal; ISAS)	4	1. Class 1/Low NSSI and functions (29%) 2. Class 2/High self-battery (32%) 3. Class 3/ High self-cutting (19%) 4. Class 4/High NSSI & functions (19%)	DV: anxiety, depression, negative self-image, somatization, and hostility (BSI) and Suicide Risk (hopelessness, hostility, suicide ideation, and negative self-evaluation (SPS)	• Class 1: Less distressed than all other classes. • Class 4: More distressed than all other classes, earlier age of onset, more frequent use of alcohol. • Class 2 and 3: Intermediate relative to the other two classes in level of distress • Class 3 and 4 were similar in their rates of past attempts which were significantly higher than proportions for Class 1 and 2
2015	Vaughn	Latent class analysis	Child sexual abuse, child physical abuse, child neglect, and family violence (binary)	4	1. Low abuse/neglect (*n* = 239; 35.57%) 2. Sexual abuse (*n* = 290; 43.15%) 3. Non-sexual abuse/neglect (*n* = 56; 8.33%) 4. High abuse/neglect and family violence (*n* = 87; 12.95%)	DV: Mental health (DSM4 clinical disorder), Substance use (AUADIS4), Crime and Violence	• Sexual abuse: More females (relative to all but High abuse) and lowest levels of all criminal and violent behaviors • Low abuse/neglect: Lowest levels of clinical and personality disorders, older than all but High abuse/neglect and family violence. • High abuse/neglect and family violence: More females (relative to all but sexual abuse) and highest levels of clinical and personality disorders, older than all but Low abuse/neglect. • Non-sexual abuse/neglect: Highest rates of alcohol, cannabis, other illicit drug use disorders, and criminal and violent behaviors. Highest levels of risk across psychosocial indices.
2013	Hamza	Latent class analysis	Lifetime frequency, most recent engagement, behavior, physical pain when injuring, amount of time between NSSI and urge, age of most recent NSSI, injuring alone (ISAS). Lifetime suicidal ideation, suicidal attempts, and disclosure (SBQR)	3	1. Low frequency NSSI/not high risk for suicidal behavior (*n* = 297; 67.7%) 2. High frequency NSSI/not high risk for suicidal behavior (*n* = 87; 19.8%) 3. High frequency NSSI/high risk for suicidal behavior (*n* = 55; 12.5%)	DV: Well-being (daily hassles, difficulties with emotion regulation [DERS], depressive symptoms [CES-D], self-esteem (RSE], social anxiety and behavioral inhibition [SASC]); Friendship quality (Inventory of Parent and Peer Attachment); Parental relationship (Inventory of Parent and Peer Attachment); Delinquency	• High frequency NSSI/high risk for suicidal behavior: Highest levels of risk across all psychosocial indices as compared other classes. • High frequency NSSI/not high risk for suicidal behavior: Lower levels of parental attachment and higher levels of parental psychological control as compared to Low frequency NSSI/not high risk for suicidal behavior.
2012	Bracken-Minor	Latent variable mixture modeling	12 NSSI behaviors, pain when injuring, injuring alone, and urgency (15 binary variables). NSSI functions (automatic and social reinforcement scales, continuous variables, ISAS)	5	1. Automatic Functions/Suicidal (*n* = 190; 43.1%) 2. Multi-Method (*n* = 139; 31.5%) 3. Experimental NSSI (*n* = 58; 13.1%) 4. Multiple Functions/Anxious (*n* = 18; 4.1%) 5. Mild NSSI (*n* = 35; 7.9%)	DV: Age of onset of NSSI, symptoms of depression and anxiety (DASS-21), BPD symptoms (MSI-BPD), hazardous drinking (AUDIT), and drinking motives (DMQ-R).	• Experimental NSSI: Lowest levels of symptoms on all clinical measures, though not significantly different from those of the Mild NSSI group; Lowest AUDIT score but not significantly different from the Mild, AF/Suicidal, or Multi-method groups. • Multi-method group had the highest levels of depression, anxiety, and BPD, followed next in severity by the AF/Suicidal group, though many times neither of these groups was significantly different from the MF/Anxious group. Highest rate of endorsement of all three suicide items, with nearly all participants in that group endorsing suicidal ideation. • Multi-function/Anxious: Highest AUDIT score. Highest scores on the coping-depression, coping-anxiety, conformity, and enhancement scales (drinking motives). • No significant difference across groups on social motives for consuming alcohol
2008	Klonsky	Latent class analysis	NSSI method, self-injuring alone or with others, functions (inter- and intrapersonal), pain, urgency (time from the urge to self-injure until the NSSI act) (ISAS)	4	1. Experimental NSSI (*n* = 125; 61%) 2. Mild NSSI (*n* = 35; 17%) 3. Multiple functions/anxious (*n* = 23; 11%) 4. Automatic functions/suicidal (*n* = 22; 10%)	DV: Depression and anxiety (DASS-21), BPD symptoms (MSI-BPD), Suicidality (YRBS), NSSI characteristics (e.g., onset)	• Mild NSSI: Earlier age of onset relative to Experimental and Automatic/functions suicidal groups. Subtypes 1 and 4. • Experimental NSSI: Lowest levels of depressive symptoms, lower levels of BPD symptoms, relative to Automatic functions/suicidal and Mild NSSI. • Multiple functions/anxious: Higher levels of anxiety symptoms than the other classes. • Automatic functions/suicidal: Greater rates of lifetime suicidal ideation, greater proportion of individuals who had attempted suicide, and higher proportion of individuals who had required medical attention for a suicide attempt, compared Experimental and Mild groups.
2008	Whitlock	Latent class analysis	Lifetime number of NSSI incidents, number of NSSI forms used, potential degree of tissue damage inflicted, age of onset, and function	3	1. Superficial NSSI (*n* = 42; 14.8%) 2. Moderate severity NSSI (*n* = 107; 37.9%) 3. High severity NSSI (*n* = 133; 47.1%)	IV: Current and past NSSI engagement, Secondary NSSI characteristics, as well as psychosocial variables and treatment history.	• High Severity NSSI: Largest proportion of individuals that currently engage in NSSI. More likely to report unintended NSSI severity, addiction, friends who self-injure, disordered eating, suicidality, and having received medication for a DSM-IV condition. More likely than Moderate Severity to report perceiving that NSSI interferes with their life, having a particular NSSI routine, injuring in phases, a history of sexual, physical, and emotional abuse and having received therapy and a clinical diagnosis.

The most common outcomes assessed between subgroups included NSSI characteristics (*n* = 23), mental health symptomology including depression (*n* = 12), anxiety (*n* = 9), borderline personality disorder (*n* = 7), emotion dysregulation (*n* = 10), and social functioning and/or relationship quality (*n* = 8; See S2 Table for a complete list of outcomes across studies). After defining the subgroups that were derived from a sample comprised only of individuals that engage in NSSI, five articles compared these subgroups to with a control group that did not have a history of NSSI [34,36–39].

### RQ1: Subgroup descriptions

Most subgroups were described along a spectrum of NSSI severity, with frequency of the behavior, tissue damage (e.g., methods used), diversification of methods, functions, and suicidality being the most common distinguishing characteristics.

#### NSSI frequency.

Measurements of NSSI frequency differed across studies, with some focusing on lifetime frequency and other focused on past year or month [33–35,40–43]. Scales also differed. Low frequency of the behavior over the course of a lifetime was typically associated with between 1-10 incidents and high frequency corresponded to over 50 incidents. In terms of past year frequency, assessments typically looked at 5 incidents as a threshold of either low or high frequency. In general, subgroups with higher frequencies of NSSI were associated with a greater level of endorsement of other NSSI characteristics including functions, motivations, methods, and suicidality. However, there were some subgroups with less frequent NSSI that also engaged in methods that could result in a high degree or tissue damage.

#### Methods.

Both method of injury and the number of methods differentiated between subgroups [31,33,35,38,43,44]. Several studies found subgroups with high rates of cutting, scratching, and burning behavior [31] or high rates of self-battery [35,38] and low endorsement of other methods. Multi-method subgroups were typically associated with earlier age of onset, more NSSI functions, and more NSSI methods that can result in tissue damage (e.g., cutting, burning) [43,44].

#### NSSI motives and functions.

Like methods, NSSI motives and functions as well as the number of motives and functions endorsed differentiated between subgroups [18,19,30,38–40,45]. Many studies looked at endorsement of intrapersonal (or automatically reinforcing functions, e.g., to regulate emotions), as compared to interpersonal functions (socially reinforcing functions; e.g., to alter the environment or social relations), as they were originally described in Nock and Prinstein’s functional model of NSSI [8]. Studies that examined these overarching functions with more granularity found that subgroups reporting greater affect regulation, anti-suicide, and multiple functions were associated with more distress and/or high rates of other NSSI characteristics (e.g., frequency) and suicidality [39,40]. Case et al., 2020 also found that self-punishment, self-care, anti-dissociation, and marking distress functions of NSSI were associated with the “Severe NSSI” subgroup.

#### Suicidality.

Several studies distinguished subgroups by suicidality [18–20,34,42,46]. In general, high suicidality groups were more likely to report three or more methods and have a history of suicidal ideation and attempt [20,34,42]. Additionally, high suicidality subgroups were associated with greater endorsement of motivations [34,46], more physical pain, and younger age of onset [20]. Contemplation before NSSI (i.e., urgency) differed across studies with Shahwan et al., 2020 finding greater contemplation and He et al., 2023 finding less contemplation, in high suicidality subgroups. Two studies identified subgroups characterized by greater endorsement of automatic/intrapersonal functions and suicidality that were also more likely to frequently injure alone [18,19]. Further, these groups were more likely to report life interference, current engagement, unintended NSSI severity, needing medical attention for injuries, and greater suicide attempt history.

#### Emotion regulation.

Studies that used emotion regulation or emotional difficulties to define subgroups differed in the types of difficulties they assessed [33,47–50]. Most studies characterized groups on a spectrum with high emotion regulation difficulties associated with other negative outcomes, including higher suicide attempt rates, negative urgency, and comorbid symptoms [33], greater past month frequency, and greater endorsement of certain functions (affect regulation, self-punishment, anti-suicide, and sensation seeking) [47]. Low emotion dysregulation groups tended to have fewer symptoms, and one study found a greater proportion of males and older individuals in the low group [47].

### RQ2: Differences in subgroup typologies by sample age, geography and analysis

Many similar typologies emerged across studies with different sample age, location, and type of analyses. We report several patterns across sample characteristics below.

#### Sample age.

Eight studies derived 22 subgroups from adolescent samples. Four studies each focused on clinical [20,37,45,50] and school [34,38,41,44] populations. The majority of these were based in the Europe [34,37,38,41,44,50], with one in Canada [45] and one in Asia (China) [20]. In general, adolescent subgroups were defined by suicidality [20,34 ] neurocognitive performance [37], social competencies [50], and NSSI method and function [38,41,44,45]. Most studies employed LCA [20,34,37,38,44,45], with two using cluster analysis [41,50].

Seven studies derived 27 subgroups from young adult samples. These were primarily drawn from universities [18,35,40,42,43,49] with one focused on a clinical sample [39]. Most studies were based in the US [18,35,40,42] with two based in Asia [39,43], and one in Canada [49]. These subgroups most often emphasized functions and methods [18,35,39,40,42,43], with one focused on emotion regulation difficulties [49]. The majority of studies used LCA [18,35,40,42], followed by cluster analysis [39,43,49]. Notably, all LCA studies were conducted in the US.

Five studies identified 19 subgroups from combined adolescent and young adult samples. Three were focused on university students [19,33,49] and two were community samples [31,32]. Most studies were US-based [19,32,33,49], with one study based in Asia (Korea) [31]. Factors defining subgroups among adolescents and young adult samples diverse, focusing on NSSI function and method [19,31], abuse history [36], emotion regulation difficulties [33], and parent/child relational styles [36]. All studies employed LCA to derive subgroups, except one study which used LVVM [19].

Finally, seven studies had age inclusive samples and identified 26 subgroups. Three were focused on university students [47,51,52], two were clinical [46,48], and one was a community sample [30]. Most studies were based in Australia [47,52,53], with one each from the US [30], Asia [46], and Europe [48]. Subgroups were defined by many factors including motivation to change [53], comorbid symptom severity [48], emotion regulation difficulties [47], personality [52], and NSSI motives and method [30,46]. Analytical procedures were mixed with many using cluster analysis [47,48,52], followed by LPA [30,53] and LCA [46].

#### Study location.

Several patterns were observed based on the geographic location of the studies. Of the eight studies based in the US, most subgroups were derived from samples of university students, with two based in the community [30,32]. There were mixed foci across groups with some focused on defining subgroups based on NSSI characteristics, and others focused on other factors (e.g., emotion regulation difficulties, abuse) and a variety of analytical approaches employed (e.g., LPA, LCA, LVVM). Notably, none of the US-based studies included on adolescent-only or clinical samples or employed cluster analysis. Of the three studies based in Australia they were all drawn from university samples without distinct age criteria [47,51,52]. They used LPA or cluster analysis, and subgroups were defined by non-NSSI factors (e.g., personality, emotion regulation difficulties, motivation). The two Canadian studies were concentrated on adolescents and young adults in a clinical [45] and university sample [49], respectively. They defined subgroups by NSSI functions and emotion regulation difficulties and used LCA and cluster analysis. The five Asia-based studies were mixed in terms of their focus on age groups (adolescent [20], adolescent and young adult [31], young adult [39,43], age inclusive [46]), and sample (clinical [20,39,46], university [43] community [31]), yet they had a primary focus on NSSI severity, function and suicidality. Finally, the seven studies from Europe were most often focused on adolescents, with one focused on an age inclusive sample [48]. Four studies were school-based [34,38,41,44], and three studies were clinical samples [37,48,51]. These studies used LCA [34,37,38,44 ] and cluster analysis [41,48,50].

#### Analysis.

In general, different analytical approaches derived similar subgroup typologies – both in number and differentiating characteristics. We note that latent class analysis was the most frequently used approach in this review. LPA analyses were exclusively applied to age inclusive datasets from the US and Australia and no US-based studies employed cluster analysis.

### RQ3: Associations between subgroups and other factors

#### Patterns in sex and gender across subgroups.

Of the 15 studies that investigated the relationship between sex or gender and subgroups, about half described differences (n=8). Most studies assessed participant sex, with fewer studies assessing gender. Just 3 studies reported participants identifying as gender minorities (i.e., transgender, non-binary, agender, genderqueer). The most consistent finding was that females were more likely to belong to subgroups with considerable suicidal ideation, relative to males [18,20,34,46]. This was true for He’s “High Suicidal Ideation NSSI” [20], DeNeve-Enthoven’s “Moderate” and “High” NSSI – High suicidality subgroups [34], Klonsky’s “Automatic functions/suicidal” subgroup [18] and Whitlocks’s “High severity NSSI” subgroup [35]. Although these subgroups were all characterized by high suicidality (suicidal ideation and/or prior attempt) and engaged in methods that involved significant tissue damage (e.g., cutting) they differed in the number of methods they endorsed (from 1 method [18] to 3 or more methods [34,35]) and the length of contemplation before self-injury (from those reporting no time between an urge and the behavior [20] to others describing more than one hour between an urge and the behavior [18]). Three of the experimental or mild subgroups (characterized by less frequent NSSI, the use of methods not likely to result in severe tissue damage, and low diversification) were also comprised of proportionately more females [20,35,43]. Among these were Whitlock’s “Superficial NSSI” subgroup [35], Singhal’s “Female Minor NSSI” subgroup [43], and He’s “Low suicidal ideation NSSI” subgroup [20]. In general, fewer subgroups were predominantly male. These subgroups were characterized by lower overall frequency of NSSI, low diversification and a greater likelihood of engaging in self-battery [35,43]. Some of these predominantly male subgroups also reported fewer emotion regulation difficulties and lower levels of abuse or neglect [32,46,47]. In general, predominantly male subgroups presented with less severe NSSI and symptomology than predominantly female subgroups.

#### Patterns in age across subgroups.

Of 18 studies that examined age across NSSI subgroups, only three studies found significant differences across subgroups. Due to variance in the characteristics used to derive and compare subgroups in these studies, it was not possible to explore how broader patterns in current age relate to NSSI heterogeneity; however, some patterns were observed for age of onset. For transparency we summarize all significant age-related differences noted in studies here. In Gray et al. (2023) subgroups were characterized based on the extent to which they wanted, or did not want, to engage in self-injury [51]. They found that the mean age of the “Avoidant” subgroup, which reported low levels of wanting to self-injure and high levels of not wanting to self-injure was older (*M* = 22.32; *range* = 21.06-23.57) than the “Approach” subgroup, (reporting high levels of wanting to self-injure, and low levels of not wanting to self-injure) (*M* = 19.94; *range* = 18.97-20.92). Shahwan et al. (2020) found that the subgroup endorsing multiple NSSI functions and low suicide ideation was older (67.57% were 21 or above vs. 20.27% 18-20 and 12.16% below 18) relative to an experimental/mild NSSI subgroup (48% were 21 or above vs. 24.44% between 18-20 and 26.67% below 18), which was characterized by low-frequency engagement in NSSI in the past year, fewer methods and a low probability of suicidality [46]. Finally, Vaughn et al., (2015) found that subgroups with the lowest (*M* = 39.17, *SD* = 15.33) and highest levels (*M* = 40.65, *SD* = 11.76) of adverse childhood events (childhood sexual abuse, physical abuse, neglect, and family violence), were younger, on average, than a subgroup with elevated levels of childhood sexual abuse (*M* = 36.30, *SD* = 12.40) and a subgroup with elevated levels of physical abuse and neglect (*M* = 37.12, *SD* = 11.26) [32].

Regarding age of onset, two studies found that Mild or Experimental subgroups were associated with earlier age of onset [18,19]. Specifically, Klonsky’s “Mild” subgroup (*M* = 11.52, *SD* = 3.14) [18] and Bracken-Minor’s “Mild” (*M* = 10.53, *SD* = 0.84) and Experimental (*M* = 11.69, *SD* = 0.69) subgroups [19] all began the behavior before the age of 12 on average, relative to other groups in the same studies that began after the age of 12 (with the exception of Klonsky’s Multiple Functions/Anxiety subgroup, *M* = 11.65, *SD* = 3.22). One study each found that higher suicidal ideation [20] and approach subgroups (a group reporting high levels of desire to self-injure, and low levels of not wanting to self-injure) [51] were also associated with earlier ages of onset with most beginning between 12-15 years of age (rather than between 16-18 years of age) in He, and an average age of onset of 13.16 years of age (*range* = 12.51-13.80), relative to onset after 14 (*Ms* = 14.17-14.81) in other subgroups in Gray [51].

#### Patterns in mental health symptoms across subgroups.

Subgroups with high levels of mental health symptomology were associated with more severe presentations of NSSI, and greater dysregulation and maladaptive coping. For example, subgroups with high rates of internalizing symptoms – such as “Sensation seeking NSSI” [39], “High NSSI and Functions” [38] and “Multiple functions/anxious” [18] - often also endorsed affect regulation functions, higher rates of suicidal ideation and suicidality, less helpful coping, more psychological distress, and difficulties with emotion regulation [18–20,30,38,39,42, 47,50 ]. These subgroups often had high levels of symptoms for depression and anxiety, rather than one being predominant over the other. Subgroups associated with high eating pathology - including the “Social Influence NSSI” [39], “High Emotion Regulation Difficulties” [33], and “High Severity” [48] subgroups - were associated with greater endorsement of mental health symptoms, emotional dysregulation, high negative urgency, and more recent NSSI. In addition having high rates of co-occurring symptoms, subgroups with high levels of substance use were often characterized by higher rates of suicide attempts [31,47] and higher rates of self-battery [19,38]. Whereas subgroups with higher BPD symptomology were associated with high suicidality, less impulsive NSSI behavior [19,39,54], and endorsement of the anti-suicide function [39].

#### Appraisal of reporting in studies.

Studies ranged in the level of detail they provided on modeling with some reporting few metrics to others reporting nearly all. On average, studies reported 9 of 17 details in the checklist (*M =* 9.42; *SD =* 2.5).. Details that were infrequently or inconsistently reported included how researchers managed missing data, the number of cases per subgroup in each model considered (and plots associated with these subgroups) and parameter restrictions or random start values as part of modeling procedures. All studies reported a distribution of observed variables (e.g., means and standard deviations; though they rarely did so for all variables that went into the derivation of subgroups, or the variables associated with subgroups), a model selection plan (though thresholds for decision making often needed to be inferred), and described the final class solution numerically. In general, most studies were missing details to allow for replication and comparability of subgroups across studies. See S3 Table for the full checklist.

## Discussion

The purpose of this review was to identify empirical studies that derived subgroups of people with a lifetime experience of NSSI, using data-driven methodologies. With regard to our research questions, we identified 26 studies that described 94 subgroups. While some studies identified subgroups with similar characteristics, and used similar naming conventions, we were unable to draw confident comparisons across studies due to variance in the samples and the ways characteristics were measured and defined (RQ1). When looking at study-level characteristics (age, geography, and type of analysis) no discernable differences were observed in the number or types of subgroups that were derived (RQ2). Finally, most studies looked at the relationship between subgroups and NSSI characteristics, comorbid mental health symptoms, and other psycho-social factors, such as history of trauma and relational functioning (RQ3). We focus the discussion on a high-level summary of patterns, research gaps and opportunities, and implications for treatment and intervention.

### Summary of findings

The studies included in this review exhibited variability in the level of methodological detail they provided which limited our ability to make robust comparisons across subgroups. Given the aims of this review, the most significant shortcoming of existing work was the absence of subgroup-level means and standard deviations for variables used to derive subgroups or outcomes. While most studies included this level of detail, some described subgroups endorsing items higher or lower relative to other subgroups within the sample which is helpful for describing typologies in a single study but limits our ability to draw more generalized conclusions. Despite this limitation, there were several robust patterns across studies that can inform treatment and intervention.

In general, there was convergence on NSSI characteristics associated with greater overall severity (i.e., comorbid symptomology, greater distress, and less effective coping or emotion regulation strategies) including endorsing more recent and frequent NSSI, a greater number of NSSI methods and functions, using at least one method with greater potential for tissue damage, experiencing pain while injuring, and greater urgency. Notably, several subgroups that were engaged in higher risk NSSI behavior reported fewer methods (often a single method) with a high degree of tissue damage (e.g., Klonsky’s and Bracken-Minor’s “Automatic Functions/Suicidal” subgroups, Singhal’s “Exclusively Severe” subgroup, and Whitlock’s “High Severity NSSI”) [18,19,35,43].

Conversely, subgroups with milder NSSI presentations (e.g., experimental [18,19, 43], mild [40,46 ], and experimental/mild subgroups [18,19,44]), were often associated with fewer co-morbid symptoms, less distress, and more effective coping strategies. They additionally engaged in NSSI methods that involve less tissue damage (e.g., banging, hair pulling, scratching), fewer forms of NSSI, and had lower endorsement of functions. While most of these subgroups engaged in less frequent NSSI relative to other subgroups from the same study, frequencies differed significantly across studies. In studies that identified both a mild and experimental subgroup, these subgroups differed in their levels of mental health symptomology and frequency of NSSI, with mild subgroups having greater symptomology, earlier age of onset and more frequent NSSI [18,19]. Of note, regardless of the level of severity of NSSI, similar subgroups were identified across clinical and non-clinical samples. For example, although most of the milder subgroups were identified in studies of university samples of young adults [18,19,40,43], several came from in clinical samples [34,47, 49].

Regarding other psychosocial factors, the findings from this review are consistent with those of a previous review, which identified psychological distress—manifested through comorbid symptoms and coping difficulties—as a key factor that distinguished between subgroups [22]. For example, subgroups that were characterized by greater difficulties social-emotional difficulties (emotion dysregulation and poorer relationship quality) often more strongly endorsed intrapersonal functions (particularly affect regulation, self-punishment), more recent and frequent NSSI, more methods, comorbid symptoms, impulsivity, and suicidality. Conversely, subgroups with less social-emotional difficulties were associated with more adaptive coping styles, and less recent, frequent and severe NSSI.

#### Gaps and opportunities.

While many of the findings from this review align with extant literature on what constitutes higher risk and severe presentations of NSSI, it is notable that few studies included the proposed DSM-5 criteria for NSSI Disorder to derive groups. Since the DSM-5 criteria are relevant for treatment planning, we feel this is an area in need of more research attention. While many studies used NSSI frequency to differentiate between classes, few looked at frequency in the past year (Criterion A), and frequency was not consistently associated with more severe presentations across subgroups. This finding reflects the lack of agreement with the DSM-5 past year criterion in the broader literature [55,56]. Additionally, several studies examined preoccupation with engaging in NSSI through the time between a NSSI thought and the behavior [19,34,41,42,44, 54], but none examined how classes differed in NSSI thoughts or experiences of interpersonal difficulties prior to injuring (Criterion C). These two variables may be important indicators of severity, and targets for intervention that merit further study. NSSI expectancies (Criterion B) were also not considered in the creation of the subgroups for any of the studies, likely reflecting the criticism on the value of this criterion in the literature [57]. Functional impairment or consequences (Criterion E) of NSSI were also not been included in any of the studies, despite being important indicators of change and useful for identifying if intervention is warranted. The remaining criteria, which are exclusionary in the DSM, were reported on inconsistently across studies (Criterion D-not socially sanctioned and F-occurs independent of other diagnoses). Interestingly factors such as pain while injuring, injuring alone, and scaring differentiated between groups across multiple studies, but are not considered as part of the proposed diagnostic criteria. These are promising additions for assessments of clinical severity, that can help guide tailored interventions.

## Implications for treatment and intervention

The results of this review enhance our understanding of NSSI heterogeneity and may be used to guide research and treatment aimed at developing personalized interventions for individuals that engage in NSSI, as well as training programs to increase confidence and competency for individuals in positions to intervene. As noted above, the most robust and consistent patterns across studies were related to the severity of NSSI presentations. Comorbidities and functions also had significant influence on subgroup membership, and merit careful attention in treatment and intervention.

On the one end of the spectrum, experimental and mild NSSI subgroups had the lowest level of symptoms. Individuals with this presentation likely do not require NSSI-specific treatment beyond psychoeducation and monitoring of NSSI behavior, and possibly development or enhancement of emotional regulation strategies to prevent reliance on NSSI in the future. Given that NSSI has been shown to be responsive to transdiagnostic interventions, such as the Unified Protocol [58], individuals identified in the experimental or mild groups may be responsive to broad treatments for emotional disorders rather than treatments designed specifically to address NSSI, such as Emotion Regulation Therapy [59,60] or Treatment for Self-Injurious Behaviors [61]. When intervening with individuals with milder symptoms, it could be helpful to note the subtle differences between those that has not yet reached a point where NSSI has become habitual or a primary way of coping (e.g., experimental subgroups), and those that do display some reliance on the behavior (e.g., mild subgroups).

For individuals with a higher level of symptoms and more severe presentations of NSSI, a treatment approach that focuses on the behavior and cognitive and behavioral strategies to address the underlying function is necessary, but likely insufficient [62,63]. More severe subgroups often presented with other comorbid symptoms that can complicate or reinforce the behavior. Treatment and intervention approaches should address comorbidities. Subgroups that experience BPD symptomology may require more specific interventions to address interpersonal or identity instability, whereas those with disordered eating may benefit from a focus on body image, for example.

For individuals reporting both high rates of suicidal ideation and current NSSI, treatments must address suicidality while considering the distinct functions underlying NSSI and suicidal behaviors. Research indicates that treatments targeting suicidality are more effective for suicidal ideation than those that do not [62]. Notably, more severe NSSI presentations showed variation, making early and comprehensive assessment important. In subgroups that report infrequent NSSI but with methods resulting in a high tissue damage, the behavior may serve as a suicidal practice rather than a regulatory mechanism. However, if assessing frequency and broad functions alone, this group may appear lower risk than they are. We note that the anti-suicide and self-punishment functions seem more common among more severe subgroups. Additionally multi-function subgroups were typically engaging in higher risk methods with more suicidality.

This review emphasizes the complexities of NSSI and the importance of thorough and comprehensive assessment in treatment and intervention. The use of diverse assessments across studies, combined with the lack of a universally accepted criteria for NSSI disorder, creates significant challenges for providers. Training programs that equip providers with a deeper understanding of NSSI, including its varied presentations and features that may indicate more severe or high-risk forms of the behavior, are promising avenue for improving identification and intervention of NSSI.

Another promising application of these findings is for the development of digital interventions. Digital interventions are increasingly common for self-injurious thoughts and behaviors as they overcome barriers to traditional treatment like availability of clinicians, costs, geography, and concerns around treatment like stigma. Until recently, however, most digital interventions have simply translated manualized treatments into internet-based formats and have not leveraged the many affordances of digital platforms for personalization and optimization. This review highlights certain characteristics that are associated with distinct clinical profiles that may be leveraged to adapt interventions or develop different intervention regimens while still adhering to evidence-based practice principles.

## Limitations

Findings from this review should be considered in the context of several limitations. First, the studies in this review are mostly focused on younger individuals (consistent with the literature and prevalence) so subgroups may not generalize to other older adults. Second, we did not assess the methodological quality or risk of bias of studies in this review. Given that many studies provided insufficient detail on the analytical procedures used to derive groups and on the magnitude of difference on factors between groups (e.g., means and standard deviations), a quality assessment may be worthwhile in future work. Thirdly, the lack of lack of standardization of timeframe for assessing NSSI characteristics, as well as studies not reporting the timescale they used for inclusion and group formation, makes it difficult to compare subgroups across studies. Fourthly, our search focused on latent class analysis and cluster analysis and thus, our sample may have missed studies using other data-driven approaches to identify subgroups such as principal components analysis. Relatedly, cluster and latent class analyses have limitations and rely on different assumptions [64]. While these models group people with homogeneous features, they do not eliminate within group heterogeneity. Groups derived from these modeling techniques show similar response patterns on the variables entered into the model, so results are inherently limited [65]. For a more comprehensive understanding of NSSI heterogeneity, future work may compare results from this review to studies that use other methodological approaches for identifying subgroups. Results from this review should be interpreted with these limitations in mind.

## Conclusion

The findings from this systematic review underscore the importance of considering NSSI heterogeneity in treatment and intervention. In general, studies identify varying levels of severity of individuals with NSSI, underscoring the importance of characteristics of NSSI in the overall severity of presenting problems. Future research using data-driven methodologies to derive subgroups of people with NSSI should closely follow quality guidelines, consider use of a standard timeframe and metrics for NSSI characteristics (e.g., number of days in the past month), and the inclusion of a broad range of mental health factors (e.g., comorbid diagnoses, BPD traits, suicidal thoughts and behaviors). Intervention research may also benefit from using common grouping characteristics derived from these studies to develop and evaluate interventions that are highly personalized and adaptive, and if intervention response varies across subgroups.

## Supporting information

S1 Appendix Search strategy. (DOCX)

S2 Appendix Decision tree for inclusion/exclusion at the title abstract phase. (DOCX)

S1 Table Features of subgroups. (DOCX)

S2 Table Outcome variables. (DOCX)

S3 Table Quality assessment results based on items from the Guidelines for Reporting on Latent Trajectory Studies (GRoLTS) checklist. (DOCX)

S4 Table Data extraction. (DOCX)

S5 Table Articles excluded in the full text review. (DOCX)

S1 PRISMA Checklist PRISMA Checklist. (DOCX)
