# Supplementary material for: Identifying subgroups of nonsuicidal self-injury: A systematic review
Source: PLOS Ment Health. 2025 Apr 21;2(4):e0000291. doi: 10.1371/journal.pmen.0000291 (PMC12363450; doi:10.1371/journal.pmen.0000291)
Supplement: S1 Appendix — (DOCX) [file pmen.0000291.s001.docx]

**S1 Appendix:** Search strategy

**PubMed**

("Self-Injurious Behavior"[Mesh] OR self-injur*[tiab] OR selfinjur*[tiab] OR self-harm*[tiab] OR selfharm*[tiab] OR self-destruct*[tiab] OR parasuicide[tiab] OR self-mutilat*[tiab] OR deliberate self-harm[tiab])

AND ("Latent Class Analysis"[Mesh] OR latent[tiab] OR cluster analysis[tiab] OR typolog*[tiab])

**MEDLINE (OVID)**

(Self-Injurious Behavior or self-injur* or selfinjur* or self-harm* or selfharm* or self-destruct* or parasuicide or self-mutilat*).ab,ti.

AND

(latent class analysis or latent or cluster analysis or typolog*).ab,ti.

*Searched with combine function.

**EMBASE**

'self injur*':ab,ti OR selfinjur*:ab,ti OR 'self harm*':ab,ti OR selfharm*:ab,ti OR 'self destruct*':ab,ti OR parasuicid*:ab,ti OR 'self mutilat*':ab,ti

AND

'latent':ab,ti OR 'cluster analysis':ab,ti OR 'typolog*':ab,ti

*Searched with combine function.

**PsychINFO**

MA self-injurious behavior OR TI ( self-injur* OR selfinjur* OR self-harm* OR selfharm OR self-destruct* OR parasuicide OR self-mutilat* ) OR AB ( self-injur* OR selfinjur* OR self-harm* OR selfharm OR self-destruct* OR parasuicide OR self-mutilat* )

MA latent class analysis OR TI ( latent OR cluster analysis OR typolog* ) OR AB ( latent OR cluster analysis OR typolog* )

(MA latent class analysis OR TI latent OR cluster analysis OR typolog* OR AB latent OR cluster analysis OR typolog*) AND (S1 AND S2)

**Web of Science**

(((TI=(self injur* OR selfinjur*OR self harm* OR selfharm* OR self destruct* OR parasuicid* OR self mutilat*')) OR AB=(self injur* OR selfinjur*OR self harm* OR selfharm* OR self destruct* OR parasuicid* OR self mutilat*')) AND AB=(latent OR cluster analysis OR typolog*)) AND TI=(latent OR cluster analysis OR typolog*)

**Scopus**

( ( TITLE-ABS-KEY ( self-injur* OR selfinjur* OR self-harm* OR selfharm* OR self-destruct* OR parasuicide OR self-mutilat* ) AND TITLE-ABS-KEY ( latent OR cluster AND analysis OR typolog* ) ) )
