# Supplementary material for: Identifying subgroups of nonsuicidal self-injury: A systematic review
Source: PLOS Ment Health. 2025 Apr 21;2(4):e0000291. doi: 10.1371/journal.pmen.0000291 (PMC12363450; doi:10.1371/journal.pmen.0000291)
Supplement: S2 Appendix — (DOCX) [file pmen.0000291.s002.docx]

**S2 Appendix:** Decision tree for inclusion/exclusion at the title abstract phase

1. Does the title/abstract mention “self-injury” or “self harm” or “self-injurious thoughts or behaviors” or “nonsuicidal self-injury”?
   - Yes → Proceed to B
   - No → Exclude
   - Unclear → Proceed to B, add note and select maybe for closer review.
2. Does the study derive subgroups, classes, groups, profiles or typologies? *
   - Yes → Proceed to C and tag
   - No → Exclude
   - Unclear → Proceed to C, add note and select maybe for closer review

** If the analysis is a “trajectory analysis” also include tag “Trajectory analysis - possibly relevant”*

1. Does the study differentiate between suicidal and nonsuicidal self-injury (study measures NSSI)?
   - Yes → Tag w/ Type 2, Proceed to D
   - No → Tag w/ Type 1, Exclude
   - Unclear → Proceed to C, add note and select maybe for closer review
2. Does the study focus on a NSSI sample (or do all participants in the sample report/have a history of NSSI)
   - Yes → Replace Type 2 tag w/ Type 3 tag, Include
   - No → Exclude (these studies should already be tagged with Type 2)
   - Unclear → Add note and select maybe for closer review

Tags:

- Type 1: Study does not differentiate between suicidal and nonsuicidal self-injury
- Type 2: Study differentiates between suicidal and nonsuicidal self-injury but the sample used for deriving subgroups was not an NSSI sample (in other words, some individuals do not engage in NSSI)
- Type 3: Study differentiates between suicidal and nonsuicidal self-injury and all individuals in the sample used to derive subgroups report engaging in NSSI.
