# Supplementary material for: Identifying subgroups of nonsuicidal self-injury: A systematic review
Source: PLOS Ment Health. 2025 Apr 21;2(4):e0000291. doi: 10.1371/journal.pmen.0000291 (PMC12363450; doi:10.1371/journal.pmen.0000291)
Supplement: S1 Table — (DOCX) [file pmen.0000291.s003.docx]

**S2 Table:** Outcome variables

|  |  |  | Mental health | | | | | | | | Emotional/Cognitive processes | | | | Social factors | | |  |  |
| --- | --- | --- | --- | --- | --- | --- | --- | --- | --- | --- | --- | --- | --- | --- | --- | --- | --- | --- | --- |
| Year | Author | NSSI characteristics | Mental health (General) | Depression | Anxiety | BPD | ED | PTSD | Substance use | Psychological distress | Emotion dysregulation | Experiential Avoidance | Self-oriented cognitions | Body-orientation/investment | Social functioning/relationship quality | Social support | Attachment style | Treatment | Demographics |
| 2023 | Gray | X |  |  |  |  |  |  |  | X | X |  |  |  |  |  |  |  |  |
| 2023 | He | X |  | X |  |  |  |  |  |  |  |  |  |  |  | X |  |  |  |
| 2023 | Kim | X | X |  |  | X |  | X |  |  |  |  |  |  |  |  |  | X |  |
| 2023 | deNeve-Enthoven | X |  |  |  |  |  |  |  |  | X |  | X |  | X | X |  |  |  |
| 2023 | Yan | X |  | X | X |  |  |  |  |  |  |  |  |  | X | X |  | X |  |
| 2022 | Dixon-Gordon | X |  | X | X | X |  |  |  | X |  |  |  |  |  |  |  |  |  |
| 2022 | Gonçalves | X |  |  |  |  | X |  |  |  | X |  |  |  |  |  |  |  |  |
| 2022 | Mürner-Lavanchy | X | X |  |  | X |  |  |  |  |  |  |  |  |  |  |  |  |  |
| 2022 | Raffagnato |  |  |  |  |  |  |  |  |  | X |  |  |  |  |  |  |  |  |
| 2022 | Reinhardt | X |  |  |  |  |  |  |  |  |  |  | X |  |  |  |  |  | X |
| 2022 | Sack | X |  |  |  | X |  |  |  |  |  |  |  |  |  |  |  |  | X |
| 2021 | Christoforou | X |  | X | X | X | X |  | X |  | X |  | X |  |  |  |  |  |  |
| 2021 | Goddard | X |  | X | X |  |  |  |  |  | X | X |  |  |  |  |  |  |  |
| 2021 | Guérin-Marion | X |  |  |  |  |  |  |  |  | X |  | X |  | X | X |  |  |  |
| 2021 | Singhal | X |  | X | X |  |  |  |  |  | X | X | X |  |  | X | X |  |  |
| 2020 | Case | X |  | X |  |  |  |  |  |  |  |  | X | X | X | X |  |  |  |
| 2020 | Shahwan | X |  | X |  |  |  |  |  |  | X |  |  |  | X |  |  |  |  |
| 2019 | Gargiulo | X |  |  |  |  |  |  |  |  | X |  |  | X |  |  |  |  |  |
| 2019 | Peterson | X |  |  |  |  | X |  | X |  | X |  |  |  |  |  |  |  |  |
| 2016 | Martin | X |  |  |  |  |  |  |  |  |  |  |  |  | X |  | X |  |  |
| 2015 | Somer |  |  | X | X |  |  |  |  |  |  |  |  |  |  |  |  |  |  |
| 2015 | Vaughn |  | X |  |  |  |  |  | X |  |  |  |  |  |  |  |  |  |  |
| 2013 | Hamza | X |  | X | X |  |  |  |  |  | X |  |  |  | X |  | X |  | X |
| 2012 | Bracken-Minor | X |  | X | X | X |  |  | X |  |  |  |  |  |  |  |  |  |  |
| 2008 | Klonsky | X |  | X | X | X |  |  |  |  |  |  |  |  |  |  |  |  |  |
| 2008 | Whitlock | X |  |  |  |  | X |  |  |  |  |  |  |  | X |  |  |  |  |
|  |  | 23 | 3 | 12 | 9 | 7 | 4 | 1 | 4 | 2 | 11 | 2 | 6 | 2 | 8 | 6 | 3 | 2 | 3 |
