# Supplementary material for: Identifying subgroups of nonsuicidal self-injury: A systematic review
Source: PLOS Ment Health. 2025 Apr 21;2(4):e0000291. doi: 10.1371/journal.pmen.0000291 (PMC12363450; doi:10.1371/journal.pmen.0000291)
Supplement: S2 Table — (DOCX) [file pmen.0000291.s004.docx]

| **S3 Table:** Results from the Guidelines for Reporting on Latent Trajectory Studies (GRoLTS) checklist | | | | | | | | | | | | | | | | | | |
| --- | --- | --- | --- | --- | --- | --- | --- | --- | --- | --- | --- | --- | --- | --- | --- | --- | --- | --- |
| Author/Year | Checklist total | Reports missing data mechanism | Describes variables related to missing data | Describes how missing data dealt with | Distribution of the observed variables reported | Software mentioned | Parameter restrictions reported | Details on covariates described (if applicable) | Random start values and final iterations reported | Model selection tools described statistically | Number of fitted models reported, including 1-class | Number of cases per class reported for **final model** | Number of cases per class reported for **each model** | Entropy reported | Plots/bar charts included for the **final solution** | Plots/bar charts included for **each model** | Final class solution numerically described | Syntax files available |
| Yan 2023 | 6 |  |  |  | X | X |  |  |  | X |  | X |  |  | X |  | X |  |
| Whitlock 2008 | 9 | X |  | X | X | X | X | X |  | X |  | X |  |  |  |  | X |  |
| Vaughn 2015 | 9 |  |  |  | X | X |  | X |  | X | X | X |  | X | X |  | X |  |
| Somer 2015 | 8 |  |  |  | X | X | X | X |  | X | X | X |  |  |  |  | X |  |
| Singhal 2021 | 8 | X | X | X | X | X |  | X |  | X |  | X |  |  |  |  | X |  |
| Shahwan 2020 | 11 | X | X | X | X | X |  | X |  | X | X | X |  | X |  |  | X |  |
| Sack 2022 | 11 |  |  |  | X | X | X | X |  | X | X | X | X | X | X |  | X |  |
| Reinhardt 2022 | 14 | X | X | X | X | X | X | X | X | X | X | X |  | X | X |  | X |  |
| Raffagnato 2022 | 8 | X |  |  | X | X |  |  |  | X | X | X |  |  | X |  | X |  |
| Peterson 2019 | 11 | X |  | X | X | X |  |  |  | X | X | X |  | X | X |  | X | X |
| Mürner-Lavanchy 2022 | 9 |  |  |  | X | X |  | X |  | X | X | X |  | X |  |  | X | X |
| Martin 2016 | 12 | X | X | X | X | X | X | X |  | X | X | X |  | X |  |  | X |  |
| Klonsky 2008 | 6 |  |  |  | X | X |  |  | X | X |  |  |  | X |  |  | X |  |
| Kim 2023 | 9 |  |  |  | X | X |  | X |  | X |  | X | X | X | X |  | X |  |
| He 2023 | 10 |  |  |  | X | X |  |  | X | X | X | X |  | X | X |  | X | X |
| Hamza 2013 | 11 | X | X | X | X | X |  |  |  | X | X | X |  | X |  |  | X | X |
| Guérin-Marion 2021 | 12 | X | X | X | X | X |  | X |  | X | X | X |  | X | X |  | X |  |
| Gray 2023 | 12 | X | X | X | X | X | X |  |  | X | X |  |  | X | X |  | X | X |
| Gonçalves 2022 | 6 |  |  |  | X | X |  |  |  | X |  | X |  |  | X |  | X |  |
| Goddard 2021 | 5 |  |  |  | X |  |  |  |  | X |  | X |  |  | X |  | X |  |
| Gargiulo 2019 | 5 |  |  |  | X | X |  |  |  | X |  | X |  |  |  |  | X |  |
| Dixon-Gordon 2022 | 13 | X | X | X | X | X |  |  |  | X | X | X | X | X | X |  | X | X |
| deNeve-Enthoven 2023 | 12 | X | X | X | X | X | X |  |  | X | X | X |  | X |  |  | X | X |
| Christoforou 2021 | 9 | X | X | X | X |  |  |  |  | X |  | X |  |  | X |  | X | X |
| Case 2020 | 8 |  |  |  | X | X | X |  |  | X | X | X |  | X | X |  | X |  |
| Bracken-Minor 2012 | 11 |  |  | X | X |  |  | X |  | X | X | X | X | X | X | X | X |  |
| Total yes | -- | 13 | 10 | 13 | 26 | 23 | 8 | 12 | 3 | 26 | 17 | 24 | 4 | 17 | 16 | 1 | 26 | 8 |
| % yes | -- | 0.5 | 0.38 | 0.5 | 1 | 0.88 | 0.31 | 0.46 | 0.12 | 1 | 0.65 | 0.92 | 0.15 | 0.65 | 0.62 | 0.04 | 1 | 0.31 |
