# Supplementary material for: Identifying subgroups of nonsuicidal self-injury: A systematic review
Source: PLOS Ment Health. 2025 Apr 21;2(4):e0000291. doi: 10.1371/journal.pmen.0000291 (PMC12363450; doi:10.1371/journal.pmen.0000291)
Supplement: S4 Table — (DOCX) [file pmen.0000291.s006.docx]

| **S4 Table.** Data Extraction | | | | | | | | | | | | | |  |
| --- | --- | --- | --- | --- | --- | --- | --- | --- | --- | --- | --- | --- | --- | --- |
| Study | Reviewer initials | Study design | Population | Geographic region | Analysis | Features | Covariates/Correlates | Auxiliary variables | Measure of NSSI | # of groups | Percentages by subgroup | Subgroup descriptions | Date | Eligible? |
| Bracken-Minor 2012 | KPK  IR | Cross-sectional | University | United states | LVMM | 15 dichotomous variables: 12 NSSI behaviors, endorsement of the absence of pain at the time of a self-injurious act, whether the individual is typically alone at the time of the act of NSSI, and whether or not the individual waited at least an hour between the urge to self-injure and the act. 2 continuous variables were included in the analysis: the automatic and social reinforcement scales of the ISAS that ranged from 0 (no endorsement of items on the scale) to 8 (maximum endorsement of the items on the scale) | age, gender, race, relationship status, time since last episode of NSSI | age of onset of NSSI, symptoms of depression, anxiety, BPD, hazardous drinking, and drinking motives. | ISAS | 5 | Automatic Functions/Suicidal 43.2%  Multi-Method 31.6%  Experimental NSSI 13.2%  Multiple Functions/Anxious 4.1%  Mild NSSI 8% | Automatic Functions/Suicidal: High probability of cutting and moderate levels of banging or hitting oneself, with low levels of social reinforcement but high levels of automatic reinforcement who almost always self-injure when alone.  Multi-Method: Moderate-to-extremely-high levels of all NSSI behaviors with the exception of swallowing dangerous substances, but otherwise mimicked the AF/Suicidal group.  Experimental NSSI: Low probabilities of most NSSI methods, with the exception of moderate levels of banging or hitting oneself, hair pulling, biting, and wound picking, with low levels of social and automatic reinforcement.  Multiple Functions/Anxious: Moderate-to-high probabilities of banging or hitting oneself, hair pulling, pinching, biting, and wound picking, and who had low probabilities of self-injuring only when alone and relatively high levels of social and automatic reinforcement.  Mild NSSI: moderate probabilities of burning and rubbing skin against rough surfaces, and high-to-extremely high probabilities of banging or hitting oneself, hair pulling, pinching, biting, wound picking, and severe scratching. However, this subgroup had relatively low levels of social and automatic reinforcement | 3/17 | Yes |
| Case 2020 | KPK, EH | Cross-sectional | University | United states | Latent class analysis | Type of methods of NSSI behaviors over one’s lifetime (e.g., cutting, burning with a cigarette, burning with a lighter, carving words, carving pictures, scratching, biting, stabbing with sharp objects, banging, punching, interfering with wound healing), number of methods used over one’s lifetime, lifetime frequency rates, last year frequency rates, number of scars resulting from NSSI, amount of pain experienced when self-injuring, and the 13 functions of NSSI (affect regulation, anti-suicide, interpersonal boundaries, self-punishment, self-  care, anti-dissociation/feeling-generation, sensation-seeking, peer-bonding, interpersonal influence, toughness, marking distress, revenge, and autonomy) |  | Self-esteem (Rosenberg Self-Esteem Scale); Body Investment (Body Investment Scale); Social Appearance Anxiety Scale; Social Support and Belonginess (Multidimensional Scale of Perceived Social Support; MSPPS) | DSHI | 4 | Mild/experimental NSSI (39%); Moderate NSSI (29%); Moderate Multiple Functions NSSI (8%); Severe NSSI (24%) | Mild/experimental NSSI: Low life- time frequency of NSSI behaviors and low number of NSSI methods employed, and compared to the sample mean, had low last year frequency rates, low scar presence, low levels of pain experienced during self-injury, and low levels of identification with ISAS functions)  Moderate NSSI: Endorsed cutting as a primary NSSI method and on average used one to two methods, and compared to the sample mean, had slightly elevated lifetime and last year frequency rates of NSSI, low scar presence, higher levels of pain experienced during self-injury, and lower levels of identification with ISAS functions, excluding affect regulation.  Moderate Multiple Functions NSSI: Endorsed cutting as a primary NSSI behavior and on average used two methods, and compared to the sample mean, had slightly elevated lifetime and last year frequency rates of NSSI, slightly higher scar presence, lower levels of pain experienced during self-injury, and significantly greater levels of identification with all ISAS functions.  Severe NSSI: Endorsed cutting as a primary NSSI behavior and on average used three methods, and compared to the sample mean had very high lifetime and last year frequency rates, very high scar presence, very high levels of pain experienced during self-injury, and greater levels of identification with specific ISAS functions, including affect regulation, anti-suicide, self-punishment, self-care, anti-dissociation, and marking distress. | 3/17 | Yes |
| Christoforou 2021 | KPK  IR | Cross-sectional | University | Australia | Cluster analysis | Emotion regulation (DERS total score, all subscales of the CERQ), Coping strategies (3 subscales of COPE), and Alexithymia (TAS-20 total score) |  | NSSI functions, Other dysregulated behaviors [Risky drinking (AusAUDIT), BPD symptoms (Borderline Symptom List), Disordered Eating (EAT-26)], Anxiety, Depression, Stress (DASS-21) | ISAS | 3 | See below | Cluster 1: (n=98) considerate diffi­culties in emotion regulation, use of less helpful coping strategies, and high levels of alexithymia.  Cluster 2: (n=78) less difficulty regulating their emotions and used more adaptive coping strategies as a response to negative emotions, focused on their problems and emotions, accepted their emo­tions, reappraised the situation, put problems it into perspective and  focused on planning a solution to their difficulties.  Cluster 3: (n=94) general difficulty in regulating their emo­tions, a lack of any of the assessed coping strategies, and moderate levels of alexithymia. | 3/17 | Yes |
| deNeve-Enthoven 2023 | KPK  IR | Cross-sectional | Primary/Secondary School | Netherlands | Latent class analysis | Lifetime NSSI frequency, lifetime number of NSSI methods endorsed, NSSI methods (cutting, biting, burning, carving, pinching, pulling hair, scratching, banging or hitting, rough surfaces), NSSI urgency, pain during NSSI, lifetime suicidal ideation, lifetime suicide attempt (all categorical), and intrapersonal and interpersonal functions of NSSI (both continuous). |  | sex, age, ethnic background, household monthly income, non-verbal IQ score, internalizing and externalizing  problems, family functioning, social support from respectively family, friends and significant others, and self-esteem | ISAS | 4 | Low NSSI–Low suicidality (n = 108, 33.6%); Moderate NSSI–Low suicidality (n = 95, 29.6%);  Moderate NSSI–High suicidality (n = 53, 16.5%);  High NSSI–High suicidality (n = 65, 20.2%) | Low NSSI–Low suicidality: Adolescents who indicated NSSI frequencies mostly in the 1–10 range (90.8%) and none had endorsed more than one method of NSSI. In this group, banging or hitting was by far the most frequently reported method (37.0%), followed by cutting (13.9%) and severe scratching (11.1%). Percentages of lifetime suicidal ideation were comparable to those reported in Class 2, with one adolescent reporting a lifetime suicide attempt. Compared to other classes, adolescents in this class recognized themselves least in statements on motivational background for performing NSSI.  Moderate NSSI–Low suicidality: Adolescents who reported NSSI frequencies predominantly in the 2–50 range (78.9%). They all reported having endorsed more than one NSSI method, but 84.2% did not endorse more than three. The most frequently endorsed methods of NSSI in Class 1 were pinching (52.6%), banging or hitting (49.5%), and biting (41.1%). None of the adolescents in this class stated to have ever cut themselves. Even though 27.4% of adolescents reported lifetime suicidal ideation, none reported a lifetime suicide attempt.  Moderate NSSI–High suicidality: The lifetime frequency of NSSI was comparable to that reported by adolescents in Mod NSSI, most  of them reported lifetime NSSI frequencies in the 2–50 range (81.1%), but none endorsed more than three methods. Cutting was reported by a large majority of adolescents (77.4%), followed by carving (50.9%) and banging or hitting (22.6%). Adolescents in this class reported high levels of suicidality; 88.5% reported lifetime suicidal ideation and 32.0% at least one suicide attempt.  High NSSI–High suicidality: Most adolescents reported NSSI frequencies in the 11–50 and >50 ranges (87.7%) and all had endorsed more than three methods of NSSI. A percentage of 81.5% of adolescents in this class reported carving, followed by banging and hitting (76.9%) and cutting (72.3%) as the second and third  most frequently endorsed methods. Lifetime suicidal ideation was reported by 92.3% and 27.7% had ever performed a suicide attempt. Compared to other classes, adolescents in this class recognized themselves the most in the statements on the ISAS-II on intrapersonal and  interpersonal motivational backgrounds for performing NSSI | 3/17 | Yes |
| Dixon-Gordon 2022 | KPK  IR | Cross-sectional; Longitudinal | Community | United states | Latent profile analysis | The five NSSI motives (i.e., emotion relief, feeling generation, interpersonal communication, interpersonal, influence, and self-punishment; from QNSSI and SASII) |  | demographic variables (i.e., age, BIPOC status, sex), NSSI characteristics (i.e., NSSI frequency, versatility [number of different NSSI methods], medical severity), and measures of psychopathology (i.e., depression, BPD, emotion regulation difficulties) as DVs. | DSHI | Sample 1: 5  Sample 2: 5 | See below | Sample 1:  (1) moderate endorsement of intrapersonal motives and low endorsement of interpersonal motives (Low Interpersonal Motives, n = 65),  (2) moderate-high endorsement of interpersonal motives and high endorsement of self-punishment motives (relative to other intrapersonal motives and other classes; Self-punishment/Interpersonal Motives, n =47),  (3) moderate intrapersonal and interpersonal motives (Moderate Intra/Interpersonal Motives, n = 19),  (4) elevated across both intrapersonal and interpersonal motives (High Intra/Interpersonal Motives, n = 14)  (5) low intrapersonal, with moderate-high interpersonal motives (Mainly Interpersonal Motives, n = 10).  Sample 2:  1) low-moderate endorsement of intrapersonal motives, and low endorsement of interpersonal motives (Low Interpersonal Motives, n = 56), (2) high endorsement of all motives (High Intra/Interpersonal Motives, n = 21),  (3) moderate-high intrapersonal motives, and low-moderate interpersonal motives (Moderate Intra/Interpersonal Motives, n = 23),  (4) low-moderate intrapersonal motives,  and high interpersonal motives (Mainly Interpersonal Motives, n = 13)  (5) high self-punishment motives, with low-moderate interpersonal motives (Self-punishment/Interpersonal Motives, n = 14). | 3/17 | Yes |
| Gargiulo 2019 | KPK  IR | Cross-sectional | Primary/Secondary School | Italy | Cluster analysis | "manifest clinical features" NSSI characteristics from the ISAS (behavior, method, lifetime frequency, past year frequency, last episode, pain, loneliness, time between thought and behavior, attempted to stop. |  | Body investment (the Body Investment Scale) and Emotion regulation (Difficulties in Emotion Regulation Strategies) | ISAS | 2 | See below | R-NSSI (N=41): adolescents who reported having self-injured from 5 to 20 times in their lives (71.4%), more than 20 times in their life (79.5%), more than 10 times in the last year (100%), those whose last episode of NSSI dated back to a few hours before the interview (75%), and those who had never tried to quit hurting themselves (61.5%). This represented the most clinically serious cluster.  E-NSSI (N=67): NSSI was used as an occasional symptom; individuals tended to scratch (81%) and hit themselves (81.58%). They had self-injured less than 5 times in their life (100%), and never (100%) or only once (100%) in the last year, those whose last episode of NSSI dated back to between 2 months and 1 year before the interview (73.8) or more than 1 year (100%), those who usually spent many hours thinking about hurting themselves  before doing it (about 80%), and those who tried at least once to stop hurting themselves (65.3%). | 3/17 | Yes |
| Goddard 2021 | KPK  IR | Cross-sectional | University | United states | Cluster analysis | Big Five personality traits |  | NSSI characteristics (ISAS), Experiential avoidance (Brief Experiential Avoidance Questionnaire), Alexithymia (Toronto Alexithymia Scale), Depression, Anxiety and Stress Scale, Emotion regulation (Cognitive Emotion Regulation Questionnaire) | ISAS | 3 | Disagreeable (N=101); Resilient (N=80); Dysregulated (N=55) | Disagreeable (Cluster I): characterized by low agreeableness and openness; tendencies towards antagonism and rigidity. Cluster I is associated with symptoms of  psychological distress within the moderate  range and ratings of alexithymia that are  higher than Cluster II.  Resilient (Cluster II): characterized by low Neuroticism and high Extraversion, Openness, Agreeableness, and Conscientiousness. It shares similarities with the resilient  prototype among people diagnosed with eat-  ing disorders.  Dysregulated (Cluster III): encompassed characteristics of both undercontrolled (low Conscientiousness and Agreeableness) and overcontrolled (high Neuroticism, low Extraversion) clusters proposed by Asendorpf et al. (2001). | 3/17 | Yes |
| Gonçalves 2022 | KPK  IR | Cross-sectional | Clinical | Portugal | Cluster analysis | Eating pathology (ED-15), emotion dysregulation (DERS) and negative urgency (UPPS-P) |  | ED-15, DERS and UPPS-P negative urgency, as well as regarding age, BMI, durations of the ED or treatment, and number of methods of NSSI, eating attitudes and dimensions of emotion dysregulation, current and past NSSI | DSM-5 | 3 | Moderate severity (N=29); High severity (N=29); Low severity (N=15) | Cluster 1 (moderate severity) was characterized  by high levels of eating pathology, but moderate emotion dysregulation and negative urgency.  Cluster 2 (high severity) was characterized by the highest scores in eating pathology, emotion  dysregulation and negative urgency, and included more patients with current non-suicidal self-injury  Cluster 3 (low severity) was characterized by the lowest levels of eating pathology, emotion dysregulation and negative urgency, and included more patients with past non-suicidal self-injury. | 3/24 | Yes |
| Gray 2023 | KPK  IR | Cross-sectional | Other: University and Community | Australia | Latent profile analysis | Extent to which one has wanted to and  not wanted to self‐injure over their lifetime. |  | Desire to self-injure and not self-injure, Tendency to approach/avoid NSSI, NSSI characteristics and functions, Personality, Reasons to stop self-injury, Difficulties in emotion regulation, Psychological distress, NSSI-related outcome expectancies, Self-Efficacy to Resist NSSI | ISAS | 4 | See below | Profile 1 (highly ambivalent; n = 30; 13.4%) reported high levels of wanting to self‐injure,  and high levels of not wanting to self‐injure throughout their lifetime.  Profile 2 (avoid; n = 39; 17.4%) reported low  levels of wanting to self‐injure, and high levels of not wanting to self‐injure throughout their lifetime.  Profile 3 (approach; n = 70; 31.3%) reported high levels of wanting to self‐injure, and low levels of not wanting to self‐injure  throughout their lifetime.  Profile 4 (moderately ambivalent; n = 85; 37.9%) reported midway levels of wanting to  self‐injure, and midway levels of not wanting to self‐injure throughout their lifetime. | 3/24 | Yes |
| Guérin-Marion 2021 | KPK  IR | Cross-sectional | University | Canada | Other: Latent cluster analysis | Self-perceived difficulties in regulating both positive and negative emotions (DERS, non-acceptance of emotions; difficulties engaging in  goal-directed behavior; impulse control difficulties; lack of emotional awareness; self-perceived limited access to strategies; and lack of emotional clarity) and Ruminative Thought Style Questionnaire | Age, gender, ethnicity, financial support, socioeconomic disparity, and present and past living arrangement (for between-profile analyses) | (1) self-reported quality of maternal and paternal relational experiences, and (2) the frequency, time elapsed since onset, methods, functions, and addictive properties of NSSI | Other: Ottawa Self-Injury Inventory | 3 | See below | Average Difficulties (Profile 1, n = 227; 85.0% female), reported lower overall difficulties than Profile 2 but higher difficulties than Profile 3 in regulating negative emotions and rumination  Dysregulated (Profile 2, n = 158; 89.2%  female) consistently had the highest self-reported difficulties in regulating negative emotions and the highest tendency toward rumination.  Low Difficulties (Profile 3, n = 94; 72.8% female) reported the lowest difficulties in regulating negative emotions, the lowest tendency toward rumination, and a lower-than-expected rate of difficulties managing positive emotions | 3/24 | Yes |
| Hamza 2013 | KPK  IR | Cross-sectional | University | Canada | Latent class analysis | NSSI variables (e.g., lifetime frequency, most recent engagement), as well as the suicidal behavior variables (e.g., lifetime suicidal ideation and suicidal attempts) |  | Well-being (daily hassles, difficulties with emotion regulation, depressive symptoms, self-esteem, social anxiety and behavioral inhibition); Friendship quality (Inventory of Parent and Peer Attachment); Parental relationship (Inventory of Parent and Peer Attachment); Delinquency (stealing money from parents/roommates, shoplifting, destroying other people’s property, impaired driving, or been the passenger in a vehicle with a driver who was impaired) | ISAS | 3 | See below | Class 1 (low frequency NSSI/not high risk for suicidal behavior; 67.7%): Characterized by lower frequency engagement in NSSI, less recent NSSI, and fewer methods of NSSI than the other two classes; lower levels of lifetime suicidal ideation/attempts, less recent suicidal ideation, and less likelihood of future attempt as compared to Class 3.  Class 2 (high frequency NSSI/not high risk for suicidal behavior; 19.8%): higher frequency of engagement in NSSI, more recent NSSI, and more methods of NSSI as compared to Class 1, but reported lower levels of lifetime suicidal ideation/attempts, lower recent suicidal ideation and lower likelihood of future suicidal attempts as compared to Class 3.  Class 3 (‘high frequency NSSI/high risk for suicidal behavior’; 12.5%): higher frequency of engagement in NSSI, more recent NSSI and more methods of NSSI than Class 1. Also reported higher levels of lifetime suicidal ideation/attempts, higher recent suicidal ideation, and greater risk for future suicidal attempts as compared to Class 1 and Class 2. | 3/24 | Yes |
| He 2023 | KPK  IR | Cross-sectional | Clinical | China | Latent class analysis | 12 NSSI variables (0: no, 1: yes) and suicidal variables (e.g., suicidal ideation, 0: no, 1: yes) |  | Suicidal attempt, depression (PHQ9), self-injury functions (FASM), Resilience (CD-RISC-10), Alexithymia (TAS), Peer victimization (MPVS) Social support (Multidimensional Scale of Perceived Social Support; MSPSS). | DSM-5 | 2 | See below | High suicidal ideation NSSI group (n=129, 39.6%): High frequency of NSSI and suicidal ideation last year, younger age of onset, more methods, high probability of suicide attempt, shorter length of contemplation before NSSI and more physical pain.  Low suicidal ideation NSSI group” (n=197, 60.4%): Low probability of NSSI, a low probability of suicidal ideation, use of "bloodless" NSSI methods. | 3/24 | Yes |
| Kim 2023 | KPK  IR | Cross-sectional / EMA | Online | Korea | Latent class analysis | Items from the self-harm inventory (SHI)- 22 behaviors (yes/no) |  | Suicide attempt (SASII), Aggression (BPAQ), Symptoms of BPD (PAI-BOR), Emotion Regulation (DERS), PTSD (IES-R)  EMA variables: Emotions, thoughts, and urges related to self-harm and suicide. | DSM-5 | 2 | See below | Substance abuse and suicide  attempt subtype (n = 30; 50%): Higher scores in overall when compared to the cutting and scratching subtype. Higher endorsement of drug abuse, suicide  attempt, and setting oneself in non-rewarding circumstances. Higher scores on overdose, cut yourself on purpose and tortured yourself with self-  defeating thoughts.  Cutting and scratching subtype (n = 30; 50%): High scores on items for Cut yourself on purpose, Hit yourself,  Scratched yourself on purpose, Tortured yourself with self-defeating thoughts, Starved yourself to hurt yourself . | 3/24 | Yes |
| Klonsky 2008 | KPK  IR | Cross-sectional | University | United states | Latent class analysis | Lifetime presence of 12 NSSI behaviors (cutting, biting, burning, carving, pinching, hair pulling, scratching, banging/hitting, wound picking, rubbing skin, needle sticking, and swallowing), descriptive features (absence of pain, whether NSSI occurs exclusively while alone, time from the urge to self-injure until the NSSI act), and two functions of NSSI (social and automatic reinforcement) |  | Depression and anxiety (DASS-21), BPD symptoms (MSI-BPD), Suicidality (YRBS), NSSI characteristics (e.g., onset) | Other: DSM-4 | 4 | See below | Experimental NSSI: (61%) performed relatively few NSSI behaviors and displayed the fewest clinical symptoms. Members of this group may be those who experimented with NSSI on a few occasions, as opposed to those who self-injure more chronically in response to psychiatric distress.  Mild NSSI: (17%) earlier onset of NSSI and performed more NSSI behaviors, particularly biting, pinching, and banging/hitting. Therefore, the NSSI in this group appears to represent more than just occasional experimentation. This group also endorsed slightly more BPD symptoms than the first group, although the overall level of clinical symptomatology was relatively low.  Multiple functions/anxious (11%): utilized a variety of NSSI methods, such as banging/hitting, biting, cutting, hair pulling, pinching, and scratching. This group also heavily endorsed both social and automatic functions, suggesting that these behaviors were multiply reinforced. Clinically, members of this group had an early onset of NSSI and displayed more symptoms of anxiety than any other group.  Automatic functions/suicidal (10%): Almost exclusively comprised those who cut themselves in private in the service of automatic functions. NSSI also appeared to be less impulsive in this group, as 60% reported that a typical instance of NSSI would occur more than 1 hr after the urge to self-injure. | 3/24 | Yes |
| Martin 2016 | KPK  IR | Cross-sectional | University | United states | Latent profile analysis | Perceptions of parent-child relational quality  (7 indicators of parent–child relationship quality were measured: maternal lack of care, maternal control, paternal lack of care, paternal control, trust in parent–child relationships, feeling alienated from parents, and relational trauma) | Age was included as a covariate in analyses regarding NSSI methods, as was current living arrangement in analyses of NSSI functions | NSSI severity and functions (The Ottawa Self-Injury Inventory) | Other: Ottawa Self-Injury Inventory (OSI) | 4 | See below | Negative - invalidating group (Profile 1: n=112): Compared to other profiles (excluding profile 4) these individuals reported more negative relational features.  Positive-moderate group (Profile 2: n=95): Rated relationships with parents positively, though not excessively so.  Positive-idealistic (Profile 3: n=35) characterized by Positive-idealistic perceptions of parent–child relationships; across all indicators, individuals within this profile reported highly positive relational quality.  Negative-disturbed (Profile 4: n=22): Significantly less trust and care from mothers, and significantly more relational trauma and alienation than all other groups | 3/24 | Yes |
| Mürner-Lavanchy 2022 | KPK  IR | Cross-sectional | Clinical | Germany | Latent class analysis | Neurocognition variables: processing speed, attention, memory, and executive functions, simple attention, visual learning, cognitive flexibility. |  | Clinical global impression, global functioning, NSSI behavior (method, behavior), BPD diagnosis and number of BPD criteria. | DSM-5 | 2 | See below | N = 176 (73.3%) patients were more likely to belong to class 1  N = 64 (26.67%) patients were more likely to belong to class 2.  Class 2 was characterized by worse neurocognitive performance on almost all tests (23 out of 24 variables). | 3/24 | Yes |
| Peterson 2019 | KPK  IR | Cross-sectional | University | United states | Latent class analysis | Frequency of different types of NSSI behavior (DSHI) and levels of different emotion regulation difficulties (DERS). |  | Acquired capability and suicide attempt history (ACSS; SHBQ); impulsive behavior (UPPS); problematic alcohol use (AUDIT) disordered (EDE-Q) | DSHI | 4 | The largest class was Class 4 (n = 238, 73%), followed by Class 2 (n = 67, 21%), Class 3 (n = 12, 4%), and Class 1 (n = 8, 2%) | Class 1 (moderate emotion regulation difficulties  with elevated frequency of cutting and burning behavior): high frequencies of cutting and burning behaviors,  moderate frequencies of banging/bruising and scratching/skin piercing  behaviors, and no endorsement of using implements.  Class 2 (high  emotion regulation difficulties with elevated frequency of scratching/  skin piercing, banging or bruising, and cutting behaviors) had high frequency of scratching/skin piercing behaviors, moderate frequencies of banging/bruising and cutting behaviors, and very low or no endorsement of burning behavior and using implements.  Class 3 (moderate levels of emotion regulation difficulties with elevated  scratching/skin piercing behaviors and low levels of using implements) had high levels of scratching/skin piercing behavior, low levels of cutting, banging/bruising behavior, and using implements, and very low or no endorsement of burning behavior.  Class 4 (low emotion regulation difficulties with low frequency of  all NSSI types): had low levels scratching/skin piercing, banging/bruising, and cutting behaviors, and very low or no endorsement of burning behavior or using implements. | 3/24 | Yes |
| Raffagnato 2022 | KPK  EH | Cross-sectional | Primary/Secondary School | Italy | Cluster analysis | The social problems, social competencies, and affective disorders scales of the Youth Self Report scale. |  | Onset of NSSI; Presence of suicidal ideation and attempt, emotional regulation (DESR); bullying, school problems, family problems, psychiatric familiarity, alcohol and substance use, traumatic  life events, and borderline intrapsychic functioning. | DSM-5 | 4 | See below. | Cluster 1/ Moderate affective difficulties with discrepant social functioning:  (n=26) some affective  difficulties, while a mixed social profile emerged because patients presented good social  competencies in conjunction with social problems.  Cluster 2 / Socio-affective impairment:  (n=21) both social and affective difficulties, since patients reported scarce social competencies  coexisting with high affective disorders and social problem  Cluster 3 / Good socio-affective functioning (n=24) characterized by a good socio-affective functioning deriving from high social  competencies and low social and affective difficulties  Cluster 4 / Low affective difficulties with discrepant social functioning: (n=23) better affective functioning, but, in a similar but different way to the previous cluster,  the social profile was not clearly defined, given that patients had low social problems  concurrently with poor social competencies | 3/24 | Yes |
| Reinhardt 2022 | KPK  EH | Cross-sectional | Primary/Secondary School | Budapest | Latent class analysis | 12 binary items of NSSI methods | NSSI motives, age, gender, perfectionism and mental health (Adolescent Mental Health Continuum) | Criterion variables: Experienced pain during NSSI, alone  during self-injury, urgency of the NSSI episode, desire to  stop NSSI | ISAS | 2 | See below | Severe/Multimethod NSSI class (39%): engaged in almost all forms of NSSI with high intensity and motivated mainly for intrapersonal reasons.  Mild/Moderate NSSI group (61%): characterized by relatively  lower risk with comparatively lower probabilities for severe  self-injury with minimal likelihood of engaging in a few  behaviors (mostly banging or hitting self and interfering  with wound healing). | 3/24 | Yes |
| Sack 2022 | KPK  EH | Cross-sectional | Clinical | Canada | Latent class analysis | NSSI functions: "feel something,” “punish yourself,” “shock/impress or get back at others,” “get away or  escape bad feelings,” “relieve anxiety/tension,” “stop feeling," self-hatred/shame,” “stop feeling anger/frustration,” “show how much you are hurting,” “regain control,” “stop frightening images,” and “create a hurt that can be soothed/healed.” |  | Gender, BPD symptoms (i.e., affective instability, negative relationships, and  impulsivity), and NSSI features (i.e., frequency and sever-  ity). | ISAS | 2 | See below | Multiple Functions class (n=28): Endorsed to “feel something,” “punish self,” “escape feelings,” “relieve anxiety,” “stop feeling self-hatred,” “stop feeling angry,” “show much they are hurting,” and “create a hurt that can be soothed."  Single/Avoidant Function class (n=40): Endorsed one primary function—  i.e., to “escape feelings." | 3/24 | Yes |
| Shahwan 2020 | KPK  IR | Cross-sectional | Clinical | Singapore | Latent class analysis | 8 indicators via FASM: (i) frequency of NSSI (ordinal), (ii) length of contemplation before  engaging in NSSI (ordinal), (iii) usage of more than three NSSI methods  (binary), (iv) suicidal ideation (binary), (v) some/often social-positive  function (binary), (vi) some/often social-negative function (binary),  (vii) some/often automatic-positive function (binary) and  (viii) some/often automatic-negative function (binary) | age, gender and ethnicity | emotion regulation (DERS), childhood trauma (CTQ), depression (PHQ8), physical and mental health (Short Form 12) | DSM-5 | 3 | See below. | Class 1—Experimental/Mild NSSI: (19.2%) Low probability of  a past suicide attempt, low probability of using more than three forms of NSSI, having high endorsement of low frequency of NSSI in last year, having high endorsement of ‘none to a  few minutes’ contemplation before NSSI and low endorsement  of all reasons/functions of NSSI.  Class 2—Multiple functions NSSI/Low Suicide Ideation: (31.5%) similar to Class 1 in terms of the low-frequency NSSI engagement, participation in fewer forms of NSSI and low probability of having had a suicide attempt. They had high endorsements on social-positive use, automatic-positive use, automatic-  negative use, and moderate endorsement on social-negative  use.  Class 3—Multiple functions  NSSI/Possible Suicide Ideation: (49.4%) characterized by high-frequency engagement in NSSI, participation in more than three forms of NSSI, high endorsement of all the functions of NSSI, that is all three  functions endorsed by Class 2 and in addition, the social-negative use of NSSI (e.g., to avoid doing something  unpleasant you do not want to do).  higher probabilities of a past suicide attempt as compared with Classes 1 and 2, higher likelihood of using more than three forms of NSSI, high probabilities of moderate  to high frequencies of NSSI, contemplating for a few minutes  or more and high endorsements on all functions for NSSI. | 3/24 | Yes |
| Singhal 2021 | KPK  IR | Cross-sectional | Community | India | Cluster analysis | Severity (based on potential degree of tissue damage by method), frequency, diversi­fication (# of NSSI methods endorsed), types of functions endorsed (interpersonal, intrapersonal, both, age of onset and gender |  | Self-criticism, brooding-rumination, emotion regulation difficulties, experiential avoidance, psychological distress, attachment style and perceived social support | DSM-5 | 5 | See below | Cluster 1 the “Multimethod NSSI” (35.70%) Members of this cluster were most likely to endorse both minor and moderate/severe methods of NSSI, more than five times in the past year with the highest diversification of NSSI. Hitting self, cutting skin, severely scratching self and pinching self were the most frequently endorsed NSSI methods, earliest onset of NSSI, higher number of functions of NSSI. he average scores on the intrapersonal functions in this  sub-group was significantly higher compared to cluster 2 and 3,  while the average scores on the interpersonal functions of NSSI was  significantly higher compared to cluster 3.  Cluster 2 “Experimental NSSI” (21%): members most likely to have re­ported between 2-4 episodes of NSSI in the past year with moderate diversification and moderate frequency. The majority reported having engaged in minor and moderate/severe methods of NSSI, with cutting skin, hitting self and severely scratching self being the most frequently endorsed NSSI methods. Onset in emerging adulthood, mostly male, lowest number of functions, lowest mean on inter and intrapersonal functions.  Cluster 3 “Female Minor NSSI” (18.10%) was most likely to endorse only minor methods of NSSI such as hitting, scratching and pinching self, with low to moderate diversification. Only females, lower number of functions compared to Multimethod group.  Cluster 4 Male Minor NSSI (13.00%)  was most likely to endorse only minor methods of NSSI predominantly hitting self, with low diversifi­cation . The majority of members of this sub-group (71.7%) reported a single episode of NSSI in the past year.  Cluster 5 Exclusively severe NSSI  (12.20%) showed endorsement of only severe methods of NSSI such as cutting, carving and burning skin. Members of this class were most likely to report a single episode of NSSI in the past year and the use of only one method of NSSI | 3/24 | Yes |
| Somer 2015 | KPK  EH | Cross-sectional | Primary/Secondary School | Turkey | Latent class analysis | 12 NSSI behaviors, 2 functions (interpersonal and intrapersonal) | BSI SPS |  | ISAS | 4 | See below | Class 1 (29 % of those who endorsed NSSI histories)  reported comparatively few NSSI behaviors and, correspondingly, had relatively low interpersonal and intrapersonal mean scores  Class 2 (32 %) was characterized by youth with relatively high likelihoods of endorsing banging or hitting behaviors (i.e., self-battery) and comparatively low probabilities associated with other NSSI behaviors.  Class 3 (19 %) had a  comparatively high likelihood of endorsing skin cutting. The mean score on intrapersonal function scale was also higher compared to Classes 1 and 2.  Class 4 (19 %)  members were likely to endorse the frequent use of a variety of NSSI behaviors, and had high scores on both interpersonal and intrapersonal function scales. | 3/24 | Yes |
| Vaughn 2015 | KPK  IR | Cross-sectional | Community | United states | Latent class analysis | Four dichotomous indicator (0 = no, 1 = yes) variables related to child maltreatment and family violence were utilized: child sexual abuse, child physical abuse, child neglect, and family violence | Mental health (DSM4 clinical disorder), Substance use (AUADIS4), Crime and Violence | Age, gender, race/ethnicity, household income, education level, and marital status | Other: DSM-4 | 4 | See below | Class 1: ‘‘low abuse/neglect’’ (n = 239; 35.57 %) characterized by individuals reporting very low levels of abuse and family violence  Class 2: ‘‘sexual abuse’’ (n = 290; 43.15 %) characterized by elevated levels of sexual abuse in concert with relatively low levels of physical abuse, parental neglect, and, to a lesser extent, family violence  Class 3: ‘‘non-sexual abuse/neglect’’ (n = 56; 8.33 %) relatively low levels of sexual abuse in concert with elevated levels of physical abuse and parental neglect, as well as elevated levels of family violence.  Class 4: ‘‘high abuse/neglect and family violence’’ (n = 87; 12.95 %) characterized by individuals who experienced adverse childhood events in all four of the domains examined | 3/24 | Yes |
| Whitlock 2008 | KPK  IR | Cross-sectional | University | United states | Latent class analysis | Lifetime number of NSSI inci-  dents, number of NSSI forms used, potential degree of  tissue damage inflicted, age of onset, and function | Gender, race, age, and SES | Current and past NSSI engagement, secondary NSSI characteristics, as well as psychosocial variables and treatment  history. | Other: DSM-4 | 3 |  | Superficial NSSI (Class 1) was composed largely of women using 1 form to engage in superficial tissue damage with moderate (<11) lifetime incidents.  Moderate severity NSSI (Class 2) was composed predominately of men using 1 to 3 forms to engage in self-battery and light tissue damage, with low (2–10) lifetime incidents.  High severity NSSI (Class 3) was composed largely of women using more than 3 self-injury forms and engaging in behaviors with the potential for a high degree of tissue damage with moderate to high numbers of lifetime incidents | 3/24 | Yes |
| Yan 2023 | KPK  IR | Cross-sectional | Clinical | China | Cluster analysis | 4 NSSI functions: Emotional regulation, Social influence, Sensation seeking, and Anti-suicide |  | Existing mental health disorders, Occasional or repeated NSSI | DSM-5 | 5 |  | Typical NSSI (Subtype I) characterized by significantly  higher scores of all four NSSI functions.  Sensation Seeking NSSI (Subtype II) characterized by significantly higher scores  of sensation seeking and high emotion regulation.  Social Influence NSSI (Subtype III)  Anti-Suicide NSSI (Subtype IV)  Untypical NSSI (Subtype V) | 3/24 | Yes |
