# Supplementary material for: Identifying subgroups of nonsuicidal self-injury: A systematic review
Source: PLOS Ment Health. 2025 Apr 21;2(4):e0000291. doi: 10.1371/journal.pmen.0000291 (PMC12363450; doi:10.1371/journal.pmen.0000291)
Supplement: S5 Table — (DOCX) [file pmen.0000291.s007.docx]

| **S5 Table:** Articles excluded in full text review | |
| --- | --- |
| **Reason for exclusion (n=34)** | **Citation** |
| Type 1: Study does not differentiate between suicidal and nonsuicidal self-injury  (*n* = 5) | Chen, W. L., & Chun, C. C. (2019). Association between Emotion Dysregulation and Distinct Groups of Non-Suicidal Self-Injury in Taiwanese Female Adolescents. *International journal of environmental research and public health*, *16*(18), 3361. <https://doi.org/10.3390/ijerph16183361>  Moniz, P., Casal, D., Mavioso, C., Videira-Castro, J., & Angélica-Almeida, M. (2011). The self-inflicted burns-Typology and its prognostic relevance in a 14-year review of self-inflicted burns in a tertiary referral centre. *Burns : journal of the International Society for Burn Injuries*, *37*(2), 322–327. <https://doi.org/10.1016/j.burns.2010.07.014>  Nesi, J., Burke, T. A., Lawrence, H. R., MacPherson, H. A., Spirito, A., & Wolff, J. C. (2021). Online Self-Injury Activities among Psychiatrically Hospitalized Adolescents: Prevalence, Functions, and Perceived Consequences. *Research on child and adolescent psychopathology*, *49*(4), 519–531. <https://doi.org/10.1007/s10802-020-00734-4>  Ricketts, E. J., Snorrason, Í., Kircanski, K., Alexander, J. R., Thamrin, H., Flessner, C. A., Franklin, M. E., Piacentini, J., & Woods, D. W. (2018). A latent profile analysis of age of onset in pathological skin picking. *Comprehensive psychiatry*, *87*, 46–52. <https://doi.org/10.1016/j.comppsych.2018.08.011>  Xin, X., Ming, Q., Zhang, J., Wang, Y., Liu, M., & Yao, S. (2016). Four Distinct Subgroups of Self-Injurious Behavior among Chinese Adolescents: Findings from a Latent Class Analysis. *PloS one*, *11*(7), e0158609. <https://doi.org/10.1371/journal.pone.0158609> |
| Type 2: Study differentiates between suicidal and nonsuicidal self-injury but the sample used for deriving subgroups was not an NSSI sample.  (*n* = 14) | Burke, T. A., McArthur, B. A., Daryanani, I., Abramson, L. Y., & Alloy, L. B. (2018). Latent classes of trait affect and cognitive affective regulation strategies are associated with depression, non-suicidal self-injury, and well-being. *Journal of Affective Disorders*, *225*, 180-187. <https://doi.org/10.1016/j.jad.2017.08.015>  Chamberlain, S. R., Redden, S. A., & Grant, J. E. (2017). Associations between self-harm and distinct types of impulsivity. *Psychiatry research*, *250*, 10–16. <https://doi.org/10.1016/j.psychres.2017.01.050>  Dhingra, K., Boduszek, D., & Klonsky, E. D. (2016). Empirically Derived Subgroups of Self-Injurious Thoughts and Behavior: Application of Latent Class Analysis. *Suicide & life-threatening behavior*, *46*(4), 486–499. <https://doi.org/10.1111/sltb.12232>  Gu, H., Yu, W., & Cheng, Y. (2024). Pattern of non-suicidal self-injury in Chinese male prisoners: A latent class analysis. *Journal of clinical psychology*, *80*(1), 86–96. <https://doi.org/10.1002/jclp.23595>  Hayes, J. A., Petrovich, J., Janis, R. A., Yang, Y., Castonguay, L. G., & Locke, B. D. (2020). Suicide among college students in psychotherapy: Individual predictors and latent classes. *Journal of Counseling Psychology, 67*(1), 104–114. [https://doi.org/10.1037/cou0000384](https://psycnet.apa.org/doi/10.1037/cou0000384)  Luyckx, K., Gandhi, A., Bijttebier, P., & Claes, L. (2015). Non-suicidal self-injury in high school students: Associations with identity processes and statuses. *Journal of adolescence*, *41*, 76–85. <https://doi.org/10.1016/j.adolescence.2015.03.003>  Marraccini, M. E., Brick, L. A., O'Neill, J. C., Weyandt, L. L., & Buchanan, A. L. (2021). Self-Injurious Thoughts and Behaviors Among College Students: A Latent Class Analysis. *Archives of suicide research : official journal of the International Academy for Suicide Research*, *25*(4), 731–750.  ​​Orlando, C. M., Broman-Fulks, J. J., Whitlock, J. L., Curtin, L., & Michael, K. D. (2015). Nonsuicidal Self-Injury and Suicidal Self-Injury: A Taxometric Investigation. *Behavior therapy*, *46*(6), 824–833. <https://doi.org/10.1016/j.beth.2015.01.002>  Polskaya, N. A., & Razvaliaeva, A. Yu. (2023). Reasons for and methods of self-harm: The results of an online survey. *Psikhiatriya*, *21*(1), 6–15.<https://doi.org/10.30629/2618-6667-2023-21-1-6-15>  Reinhardt, M., Horváth, Z., Drubina, B., Kökönyei, G., & Rice, K. G. (2021). Latent class analysis of nonsuicidal self-injury among justice-involved juveniles: Association with motivational and emotional aspects of self-harm behavior. *Criminal Justice and Behavior, 48*(7), 902–922. [https://doi.org/10.1177/0093854821998411](https://psycnet.apa.org/doi/10.1177/0093854821998411)  Sun, F., Li, H., Song, W., Bao, J., & Zhen, Z. (2022). Patterns of psychological pain and self-harm behaviors in adolescents. *Suicide & life-threatening behavior*, *52*(5), 1012–1023. <https://doi.org/10.1111/sltb.12898>  Thomassin, K., Quint, E., Sezlik, S., & Shaffer, A. (2017). Profiles of Emotion Deficits and Adolescent Nonsuicidal Self-Injury in an Inpatient Sample. *Journal of research on adolescence : the official journal of the Society for Research on Adolescence*, *27*(4), 752–764. <https://doi.org/10.1111/jora.12312>  Zelkowitz, R. L., Halverson, T. F., Patel, T. A., Beckham, J. C., Calhoun, P. S., Pugh, M. J., & Kimbrel, N. A. (2023). Nonsuicidal self-injury methods among U.S. Veterans: Latent class analysis and associations with psychosocial outcomes. *Psychiatry research*, *329*, 115558. <https://doi.org/10.1016/j.psychres.2023.115558>  Gu, H., Yu, W., & Cheng, Y. (2024). Pattern of non-suicidal self-injury in Chinese male prisoners: A latent class analysis. *Journal of clinical psychology*, *80*(1), 86–96. <https://doi.org/10.1002/jclp.23595> |
| Wrong analysis (e.g., study did not derive subgroups)  (*n* = 10) | Barrocas, A. L., Giletta, M., Hankin, B. L., Prinstein, M. J., & Abela, J. R. (2015). Nonsuicidal self-injury in adolescence: longitudinal course, trajectories, and intrapersonal predictors. *Journal of abnormal child psychology*, *43*(2), 369–380. <https://doi.org/10.1007/s10802-014-9895-4> **(Latent Trajectory Analysis)**  Czyz, E. K., & King, C. A. (2015). Longitudinal trajectories of suicidal ideation and subsequent suicide attempts among adolescent inpatients. *Journal of clinical child and adolescent psychology : the official journal for the Society of Clinical Child and Adolescent Psychology, American Psychological Association, Division 53*, *44*(1), 181–193. <https://doi.org/10.1080/15374416.2013.836454> **(Latent Class Growth Modeling)**  Dawe-Lane, E., & Flouri, E. (2023). Parenting in the early years and self-harm in adolescence: The role of control and reward systems in childhood. *Journal of affective disorders*, *339*, 788–798. <https://doi.org/10.1016/j.jad.2023.07.061> **(Latent Growth Curve Analysis)**  Gao, Q., Guo, J., Wu, H., Huang, J., Wu, N., & You, J. (2021). Different profiles with multiple risk factors of nonsuicidal self-injury and their transitions during adolescence: A person-centered analysis. *Journal of affective disorders*, *295*, 63–71. <https://doi.org/10.1016/j.jad.2021.08.004> **(Latent Transition Analysis)**  Heilbron, N., & Prinstein, M. J. (2010). Adolescent Peer Victimization, Peer Status, Suicidal Ideation, and Nonsuicidal Self-Injury: Examining Concurrent and Longitudinal Associations. *Merrill-Palmer quarterly (Wayne State University. Press)*, *56*(3), 388–419. <https://doi.org/10.1353/mpq.0.0049> **(Latent Growth Curve Analysis)**  Jones, R. S. (1987). The relationship between stereotyped and self-injurious behaviour. *British Journal of Medical Psychology, 60*(3), 287–289. [https://doi.org/10.1111/j.2044-8341.1987.tb02744.x](https://psycnet.apa.org/doi/10.1111/j.2044-8341.1987.tb02744.x) **(No statistical analysis)**  Tilton-Weaver, L., Latina, D., & Marshall, S. K. (2023). Trajectories of nonsuicidal self-injury during adolescence. *Journal of adolescence*, *95*(3), 437–453. <https://doi.org/10.1002/jad.12126> **(Latent Growth Curve Modeling)**  Voon, D., Hasking, P., & Martin, G. (2014). Change in emotion regulation strategy use and its impact on adolescent nonsuicidal self-injury: a three-year longitudinal analysis using latent growth modeling. *Journal of abnormal psychology*, *123*(3), 487–498. <https://doi.org/10.1037/a0037024> **(Latent Growth Curve Modeling)**  Wang, B., You, J., Lin, M. P., Xu, S., & Leung, F. (2017). Developmental Trajectories of Nonsuicidal Self-Injury in Adolescence and Intrapersonal/Interpersonal Risk Factors. *Journal of research on adolescence : the official journal of the Society for Research on Adolescence*, *27*(2), 392–406. <https://doi.org/10.1111/jora.12273> **(Latent Class Growth Analysis)**  Heilbron, N., & Prinstein, M. J. (2010). Adolescent Peer Victimization, Peer Status, Suicidal Ideation, and Nonsuicidal Self-Injury: Examining Concurrent and Longitudinal Associations. *Merrill-Palmer quarterly (Wayne State University. Press)*, *56*(3), 388–419. <https://doi.org/10.1353/mpq.0.0049> **(Latent Growth Curve Analysis)** |
| No English language version available  (*n* = 4) | Antretter, E., Dunkel, D., Seibl, R., & Haring, C. (2002). Die klassifikatorische und prädiktive Qualität des Merkmals "suicide intent" bei Parasuiziden: Eine clusteranalytische Untersuchung [The discriminant and predictive quality of suicide intent. A cluster-analytic approach]. *Der Nervenarzt, 73*(3), 219–230. [https://doi.org/10.1007/s001150101196](https://psycnet.apa.org/doi/10.1007/s001150101196)  Castro, K., Kirchner, T., & Planellas, I. (2014). Predicción de conducta autodestructiva en adolescentes mediante tipologías de afrontamiento [Predicting self-destructive behavior in adolescents by means of coping typologies]. *Universitas Psychologica, 13*(1), 121–133.doi:10.11144/Javeriana.UPSY13-1.pcaa  Castro, K., Kirchner, T., & Planellas, I. (2014). Predicción de conducta autodestructiva en adolescentes mediante tipologías de afrontamiento [Predicting self-destructive behavior in adolescents by means of coping typologies]. *Universitas Psychologica, 13*(1), 121–133.doi:10.11144/Javeriana.UPSY13-1.pcaa  Krylova, E. S., Kuleshov, A. A., & Kaleda, V. G. (2023). Psikhopatologicheskie korrelyaty depressii s nesuitsidal'nymi samopovrezhdeniyami pri nepsikhoticheskikh psikhicheskikh zabolevaniyakh yunosheskogo vozrasta [Psychopathological correlates of depression with non-suicidal self-harm in non-psychotic mental illnesses of adolescence]. *Zhurnal nevrologii i psikhiatrii imeni S.S. Korsakova*, *123*(11. Vyp. 2), 74–78. https://doi.org/10.17116/jnevro202312311274 |
| Not a peer reviewed article  (*n* = 1) | Medrano, J. I., Albores, L., Torres, G., & others. (2023). The Spanish Self-Injury Diagnostic Schedule: A latent class analysis. *Journal of the American Academy of Child & Adolescent Psychiatry*, *62*(10), S313–S314. |
