# Supplementary material for: Identifying subgroups of nonsuicidal self-injury: A systematic review
Source: PLOS Ment Health. Author manuscript; Available in PMC 2025 Aug 20. (PMC12363450; doi:10.1371/journal.pmen.0000291)
Supplement: Supplementary file 1 [file NIHMS2083160-supplement-S1_Table.docx]

**S1 Table:** Features of subgroups

| Year | Author | NSSI char. | Lifetime freq. | Recent freq. | Last NSSI | Functions | Motives | Methods | # of methods | Age of onset | Urgency | Pain | Scarring | Injuring alone | Suicidal ideation | Suicide Attempt | Suicide  attempt disclosure | Desire to engage/stop NSSI | Eating pathology | Depression/Alexithymia | Emotion dysregulation | Social Competencies | Rumination | Trauma | Childhood /Family abuse | Parent/ Family relationship | Coping style | Personality | Neurocognitive performance |
| --- | --- | --- | --- | --- | --- | --- | --- | --- | --- | --- | --- | --- | --- | --- | --- | --- | --- | --- | --- | --- | --- | --- | --- | --- | --- | --- | --- | --- | --- |
| 2023 | Gray |  |  |  |  |  |  |  |  |  |  |  |  |  |  |  |  | X |  |  |  |  |  |  |  |  |  |  |  |
| 2023 | He | X |  |  |  |  |  | X |  |  |  |  |  |  | X | X |  |  |  |  |  |  |  |  |  |  |  |  |  |
| 2023 | Kim | X |  |  |  |  |  | X |  |  |  |  |  |  |  |  |  |  |  |  |  |  |  |  |  |  |  |  |  |
| 2023 | deNeve-Enthoven | X | X |  |  | X |  | X | X |  | X | X |  |  | X | X |  |  |  |  |  |  |  |  |  |  |  |  |  |
| 2023 | Yan | X |  |  |  | X |  |  |  |  |  |  |  |  |  |  |  |  |  |  |  |  |  |  |  |  |  |  |  |
| 2022 | Dixon-Gordon | X |  |  |  |  | X |  |  |  |  |  |  |  |  |  |  |  |  |  |  |  |  |  |  |  |  |  |  |
| 2022 | Gonçalves |  |  |  |  |  |  |  |  |  |  |  |  |  |  |  |  |  | X |  | X |  |  |  |  |  |  |  |  |
| 2022 | Mürner-Lavanchy |  |  |  |  |  |  |  |  |  |  |  |  |  |  |  |  |  |  |  |  |  |  |  |  |  |  |  | X |
| 2022 | Raffagnato |  |  |  |  |  |  |  |  |  |  |  |  |  |  |  |  |  |  | X |  | X |  |  |  |  |  |  |  |
| 2022 | Reinhardt | X |  |  |  |  |  | X |  |  |  |  |  |  |  |  |  |  |  |  |  |  |  |  |  |  |  |  |  |
| 2022 | Sack | X |  |  |  | X |  |  |  |  |  |  |  |  |  |  |  |  |  |  |  |  |  |  |  |  |  |  |  |
| 2021 | Christoforou |  |  |  |  |  |  |  |  |  |  |  |  |  |  |  |  |  |  | X | X |  |  |  |  |  | X |  |  |
| 2021 | Goddard |  |  |  |  |  |  |  |  |  |  |  |  |  |  |  |  |  |  |  |  |  |  |  |  |  |  | X |  |
| 2021 | Guérin-Marion |  |  |  |  |  |  |  |  |  |  |  |  |  |  |  |  |  |  |  | X |  | X |  |  |  |  |  |  |
| 2021 | Singhal | X | X* |  |  | X |  | X | X | X |  |  |  |  |  |  |  |  |  |  |  |  |  |  |  |  |  |  |  |
| 2020 | Case | X | X | X |  | X |  | X | X |  |  | X | X |  |  |  |  |  |  |  |  |  |  |  |  |  |  |  |  |
| 2020 | Shahwan | X | X* |  |  | X |  | X | X |  |  |  |  |  | X |  |  |  |  |  |  |  |  |  |  |  |  |  |  |
| 2019 | Gargiulo | X | X | X | X |  |  | X |  |  | X | X |  |  |  |  |  | X |  |  |  |  |  |  |  |  |  |  |  |
| 2019 | Peterson | X | X |  |  |  |  | X |  |  |  |  |  |  |  |  |  |  |  |  | X |  |  |  |  |  |  |  |  |
| 2016 | Martin |  |  |  |  |  |  |  |  |  |  |  |  |  |  |  |  |  |  |  |  |  |  | X |  | X |  |  |  |
| 2015 | Somer | X |  |  |  | X |  | X |  |  |  |  |  |  |  |  |  |  |  |  |  |  |  |  |  |  |  |  |  |
| 2015 | Vaughn |  |  |  |  |  |  |  |  |  |  |  |  |  |  |  |  |  |  |  |  |  |  |  | X |  |  |  |  |
| 2013 | Hamza | X | X |  | X |  |  | X |  |  | X | X |  | X | X | X | X |  |  |  |  |  |  |  |  |  |  |  |  |
| 2012 | Bracken-Minor | X |  |  |  | X |  | X |  |  | X | X |  | X |  |  |  |  |  |  |  |  |  |  |  |  |  |  |  |
| 2008 | Klonsky | X |  |  |  | X |  | X |  |  | X | X |  | X |  |  |  |  |  |  |  |  |  |  |  |  |  |  |  |
| 2008 | Whitlock | X | X |  |  | X |  | X | X | X |  |  |  |  |  |  |  |  |  |  |  |  |  |  |  |  |  |  |  |
|  | Total | 17 | 8 | 2 | 2 | 10 | 1 | 13 | 5 | 2 | 5 | 6 | 1 | 3 | 4 | 3 | 1 | 2 | 1 | 2 | 3 | 1 | 1 | 1 | 1 | 2 | 1 | 1 | 1 |
